# Digital Group–Based Intervention for Physical Activity Promotion Among Thai Adults During the COVID-19 Lockdown: Randomized Controlled Trial

**DOI:** 10.2196/43366

**Published:** 2024-01-31

**Authors:** Nanthawan Pomkai, Piyawat Katewongsa, Aphichat Chamratrithirong, Kanokwan Tharawan, Teeranong Sakulsri, Bhubate Samutachak, Dyah Anantalia Widyastari, Niramon Rasri, Boonyanuch Wijarn, Yodchanan Wongsawat

**Affiliations:** 1 Institute for Population and Social Research Mahidol University Nakhon Pathom Thailand; 2 Thai Health Promotion Foundation Bangkok Thailand; 3 Popmed Co, Ltd Bangkok Thailand; 4 Department of Biomedical Engineering Faculty of Engineering Mahidol University Nakhon Pathom Thailand

**Keywords:** physical activity, digital group–based activity, collective action, Thai adults, COVID-19 pandemic, COVID-19, design, model, effectiveness, digital, Thailand, behavior

## Abstract

**Background:**

The COVID-19 pandemic significantly diminished the physical activity (PA) level of Thai adults belonging to Generation Y (Gen Y). As a response to the global crisis, many individuals worldwide have turned to social community platforms, recognizing their potential in promoting PA during the pandemic. Gen Y, in particular, demonstrates exceptional proficiency in using social media platforms, showcasing a remarkable aptitude for swiftly accessing new information and knowledge. However, their proclivity for reckless behavior exposes them to various health risks, potentially leading to enduring adverse health consequences. Consequently, there arises a pressing need to develop a comprehensive model aimed at elevating the PA levels among individuals belonging to Gen Y.

**Objective:**

This research aimed to examine the effectiveness of a digital group–based activity in promoting PA among Gen Y in Thailand.

**Methods:**

This was a parallel 2-arm randomized controlled trial with single-blind allocation to experimental and control groups and pre- and posttest measurements. Measurements were administered on the web and were designed for respondents to complete by themselves. The sample comprised 100 Gen Y individuals who met the inclusion criteria. Both groups were matched for background characteristics. The two 8-week intervention activities were (1) two weeks of education and (2) six weeks of motivation by target groups that set goals for PA together (using the Zoom meeting application), with a time limit and group consensus as to when the goal was to be achieved. The intervention activities were implemented one by one at specified intervals and delivered daily through health apps and the official LINE account.

**Results:**

The intervention starts from August 22 to October 16, 2021. Of the 100 participants, 20 (20%) left the study, and the remaining 80 (80%) participated in the study (40 individuals each in the experimental and control groups). After participating in the experiment, a statistically significant difference in PA was found between the 2 groups (moderate to vigorous PA; 25/40, 63%; *P*=.03). Participants in the intervention group collected a higher cumulative minute of moderate to vigorous PA weekly (283 minutes) than those in the control group (164 minutes), and this was statistically significant (*P*=.03). For the transition to the fourth stage of behavior (ie, action), the improvement in the experimental group, after participating in the trial, was statistically significant compared to that of the control group (*P*=.01).

**Conclusions:**

Digital group–based activity showed its effectiveness in improving the PA of Gen Y individuals in the intervention group. It created a process-based intervention activity that corresponds to the stages of behavior changes, from contemplation to action. The digital community can also connect individuals to comparable groups locally and globally.

**Trial Registration:**

Thai Clinical Trials Registry TCTR20211101005; https://www.thaiclinicaltrials.org/show/TCTR20211101005

## Introduction

The importance of physical activity (PA) for health has been documented worldwide. For adults, regular and adequate PA has been associated with a lower risk of premature deaths due to noncommunicable diseases, lower incidence of hypertension, type 2 diabetes, and cancer; improved cognitive function; and better mental health outcomes [1,2]. Nevertheless, although these benefits have been acknowledged and public health promotion efforts have been undertaken, physical inactivity remains a global public health problem [3].

The COVID-19 pandemic has forced nationwide lockdowns, and most of the countries use social distancing measures and policies to encourage people to stay at home to reduce the spread of the disease [4]. These measures have restricted people’s movement and led to a reduction in PA globally [5,6]. With limited opportunity to engage in regular outdoor PA during containment periods, lifestyle changes are required. Health promotion strategies have then focused on the message of promoting home-based PA and encouraging population of all ages to stay active while at home [7]. The World Health Organization (WHO) initiated the “Healthy at Home” campaign, and it was followed by many countries with a similar focus: encouraging home-based PA to regenerate the PA of the population [7-10].

The promotion of home-based PA was purposefully executed through social media platforms and group-based activities. This involved recommending home-based exercises; providing sample training videos; and offering free web-based exercise classes on platforms such as YouTube, Facebook, and Twitter. Access was facilitated through social media platforms or applications, with the primary goal of helping individuals maintain a healthy weight, reduce the risk of chronic diseases, and foster social interaction [2,8,10-12]. A few studies have documented the positive effect of home-based PA promotion (eg, Fit From Home in improving the PA of the population) [8,12].

While the actual community interaction was limited during the pandemic, the digital community has been increasingly demanding and becoming an everyday part of every individual’s life [13]. This community is a place for people to gather on the web, who may or may not meet one another face-to-face, to stay in touch with or perform certain activities in the groups they belong to [14]. The digital engagement creates and maintains relationships between group members in the digital group–based activity, affecting both psychological and social motivation, and this can lead to behavior modification of members within the group [8,13].

With more than half of the people globally now using social community [15], digital group–based activity (a digital community platform) has the potential to promote PA during the pandemic [8,10-12,16]. Unlike the mass campaigns that are addressed to the general population, information delivered in the digital group–based activity targeted specific groups that share similar interest. Good engagement within the group increases the effectiveness of the messages in driving the behavior changes of its members [12,17,18].

Adults who belong to the Generation Y (Gen Y) are a group of population with high literation in technology or digital native generation and are those who use IT as part of their daily life [19-21]. Gen Y in Thailand (those born between 1981 and 2000) [22-26] who have access to the internet, comprising a total of 17,644,290 (26.6%) people [16], are the most active in using the social media platform compared to other working-age groups [16]. They are exceptionally quick to access new information and knowledge and share this at lightning speed among their social networks. They are creative and have high self-esteem, and they prefer working in teams or working together in groups [27]. However, it is also the group that has the opportunity to contract various diseases due to reckless behavior, and that hazard may have lifelong adverse health consequences [28]. Therefore, it is necessary to find specific measures to encourage PA, especially for those who are undecided about changing their behavior.

Previous studies reported a high probability of behavior changes if appropriate intervention and health promotion efforts are undertaken [18]. Collective action theory has identified mechanisms to increase motivation through the creation of a common goal and by setting conditions for achieving that goal with others [29]. By providing education and motivation through a social media platform, the use of group processes should be able to induce behavioral changes and help promote the adoption of healthier lifestyles. While the collective action theory was useful in developing the model intervention, the transtheoretical model (TTM) is relevant in describing the stages of behavioral change [30,31]. This theory argues that an individual’s health behavior change can be observed by following the stages of “precontemplation, contemplation, preparation, action, maintenance, and termination” [31]. By understanding which stage an individual is in, an appropriate intervention could be tailored. Guided by the 2 theories, this study aimed to examine the effectiveness of digital group–based activity to improve Thai adults’ PA with a focus on Gen Y by considering the context of their lifestyle and habits. The findings of this study should be useful in providing information for decision-making in promoting PA of adults in the future.

## Methods

### Study Design

A parallel 2-arm randomized controlled trial design with single-blind assignment of participants to experimental and control groups was used. Outcomes were measured by pre- and posttest assessments (pre- and posttest design with the control group). Matching between the experimental and control groups was performed by aligning the individual characteristics, for example, sex, age group, and primary occupation, by using probability random sampling. While the intervention group received a set of interventions, the control group performed their routines without any intervention.

### Population and Sample

The population universe in this research is Thai adults who belong to Gen Y and who have access to the internet, or a total of 17,644,290 people. Gen Y was defined as a group of people born between 1981 and 2000 [22-26]. This segment of the population is critical for the future development of the country, both in the labor market and in terms of the economy (insofar as the Gen Y group dominates the spending power of the country) [32]. This digital population was randomly sampled (with probability known) to provide information on Thailand’s PA surveillance system. The prescribed sample size was calculated by considering the value α=.05, effect size=0.5 (a medium effect size from the previous study [33]), power=0.95, and a 2-tailed *t* test. To reduce attrition due to withdrawal from the study or refusal to participate, the sample size was increased from the minimum prescribed number (54 persons), resulting in a total sample of 100 people, evenly divided into experimental and control groups.

Eligible participants were those who met the inclusion criteria of having insufficient PA as recommended by the WHO [34] and were in the second stage of behavior change (ie, contemplation) because 1 in 3 Gen Y individual is in this stage [35]. Simple random sampling with replacement was used to randomly assign the participants to the experimental group or control group. In case of sample rejection, a suitable replacement was selected.

### Randomization

Once the sample size was known, the researcher conducted random sampling to obtain the participants in the research by concealment of allocation sequences, and participants did not know that they were assigned to the experimental or control group. The steps are as follows:

Step 1 involved screening samples to participate in the experiment. The population universe was Thai individuals of all genders and ages who had access to the internet at the time of the survey. The sample was selected through multistage random sampling. For this study, the population of interest was members of Gen Y. Eligible participants were screened according to the control variables, namely, sex, age group, and primary occupation. The inclusion criteria were those who had insufficient PA as recommended by the WHO and were in the second stage of behavior change (ie, contemplation).

In step 2, simple random sampling with replacement was used to randomly select between the experimental group and control group. At this stage, the prior random individual was assigned to the experimental group, and then, another of its pair would be assigned to the control group. This loop proceeded until all required samples were matched as planned.

In step 3, after the sampling process was completed, the consent and voluntary participation were inquired. In case of sample rejection, the new random sample with the same eligible participants and method was used for replacement.

### The Intervention: Digital Group–Based Activity

#### Overview

For the prototype model in this study, the intervention covered 8 weeks of activity (from August 22 to October 16, 2021). The designed interventions consisted of education and motivation (as per theory), with the intervention activities implemented one by one at specified intervals, delivered daily through social media platforms (health apps and the official LINE account [LY Corporation]). The “Light-hearted” app (Popmed Co Ltd) was designed as a mobile-based app considering that Gen Y individuals are the heavy users of digital media. This app recorded the number of steps and other PAs including biking, swimming, walking, boxing, volleyball, and badminton. Participants were also requested to record their daily calorie intake. Additional features including health information and feedback mechanism (ie, suggestion box) were also made available to improve participants’ engagement apart from the chat box (Table 1).

**Table 1:** The intervention program for the experimental group was sent through the health apps “Light-hearted” and LINE.

Week and topic		Activity
**Intervention 1: Providing knowledge to increase awareness**		
	Week 1: Exposed to PA^a^ education messages	PA self-assessment The importance of PA Knowledge of the PA Advantages and disadvantages of the original behavior Cobenefits of sufficient PA Dramatic relief
	Week 2: The guideline practice of various PAs	Information about alternatives to PA and PA for the working-age population (at home or around the house) Information about alternatives to PA at work and in public areas
**Intervention 2: Creating motivation**		
	Week 3-6: Collective motivation of the group by encouraging the determination to reach the set goals	Setting group goals Target groups set goals for PA together with a time limit and group consensus Start accumulating steps through the health app The group’s cumulative steps were reported every 3 days including sending motivation messages to groups

^a^PA: physical activity.

#### Providing Knowledge to Increase Awareness

This part of the intervention lasted 2 weeks. This activity aimed to build knowledge and understand the guidelines on PA for the intervention group. In the first week, participants were exposed to health education messages (infographic and video clips) sent through the health apps “Light-hearted ” and LINE. The health apps “Light-hearted” and LINE were installed after the intervention group consented to participate in the intervention. The messages covered PA self-assessment, the importance of PA, advantages and disadvantages of the original behavior, cobenefits of sufficient PA, and dramatic relief. In the second week, the health education message described the practice of various PAs. This included information about alternatives to PA and PA for the working-age population at home or around the house, at work, and in public areas. The intention was to add a variety of options for PA. The video was produced by Social Marketing Team of ThaiHealth Promotion Foundation, was in the public domain (SocialMarketingTH YouTube channel), and was free to be used. The videos contained messages on the causes of noncommunicable diseases, shared experiences, and motivation for regular exercise.

#### Creating Motivation

A total of 6 weeks was spent to apply the collective action theory, which was the main process hypothesized to drive behavior change in this study. Target groups set goals for PA together, with a time limit and group consensus as to when the goal was to be achieved. The collective action theory prescribes both internal and external motivation. Both types of motivation were directly targeted by the intervention activity and indirectly targeted through discussion and encouragement via group chat as an additional impetus to behavior change. The process started with setting group goals by using Zoom (Zoom Video Communications) meetings to discuss PA goals in the entire experimental group. Ultimately, it was agreed that all participants would accumulate steps through the health app “Light-hearted.” The group’s cumulative steps were reported every 3 days. At the end of the intervention, if the goal of the activity was achieved, then the research project would make a donation to charity as promised.

### Measurement

A structured questionnaire was used as the data collection tool. PA and sedentary behavior were assessed by using the Global Physical Activity Questionnaire (version 2), while behavior assessment used the standardized and validated TTM questionnaire [35]. Sufficient PA was calculated following the guideline that includes (1) work-related activity, (2) traveling from place to place, and (3) leisure time activity [34]. The questions were self-administered on the web in the LimeSurvey web application (LimeSurvey) and were designed appropriately for the respondents to complete by themselves (ie, PA).

### Data Analysis

Univariate analysis was used to describe the characteristics and distribution of the sample, such as percentage, mean, median, and range. Bivariate analysis was used to test for significant association of variables and group-level differences. To test the effectiveness of the model, the mean before-and-after values in the same group were compared by the paired 2-tailed *t* test, while the means between the 2 groups were compared by the independent samples 2-tailed *t* test.

### Ethical Considerations

**Figure 1:**
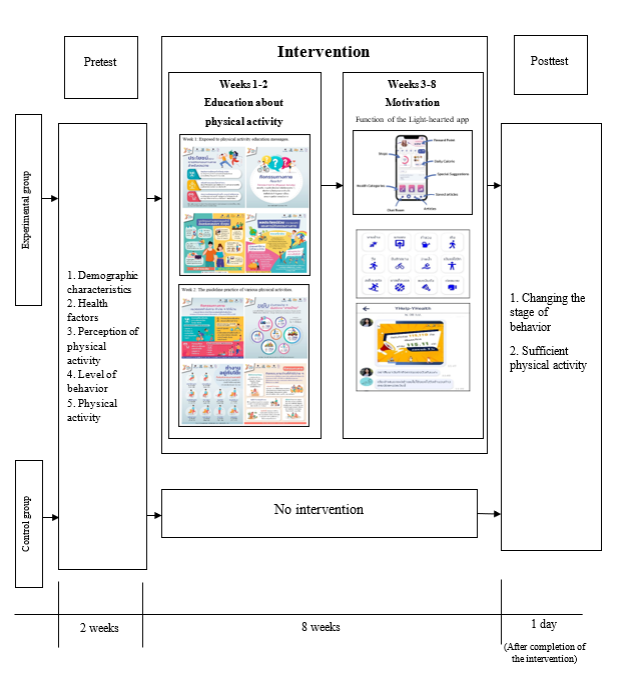
Process of the intervention of this study.

All procedures performed in studies involving human participants were in accordance with the ethical standards of the institutional and national research committee and with the 1964 Helsinki Declaration and its later amendments or comparable ethical standards. The study protocol was reviewed and approved by the Research Ethics Committee of the Institutional Review Board, Institute for Population and Social Research, and Mahidol University on April 28, 2021 (project code COA 2021/03-048) and registered on the Thai Clinical Trials Registry (TCTR20211101005). All participants agreed in advance to provide data to the researchers and were assured that all their information would be kept confidential. Participants provided their consent to be included in the study by clicking on the agreement box available in the LimeSurvey web application. No personal information of the participant was collected or used in a way that would identify the data provider. As compensation, participants in the intervention group who completed two rounds received a T-shirt valued 250 Thai Baht (US $7). If the intervention was successful in the experimental group, the intervention would also be offered to the control group participants (Figure 1).

## Results

### Overview

**Figure 2:**
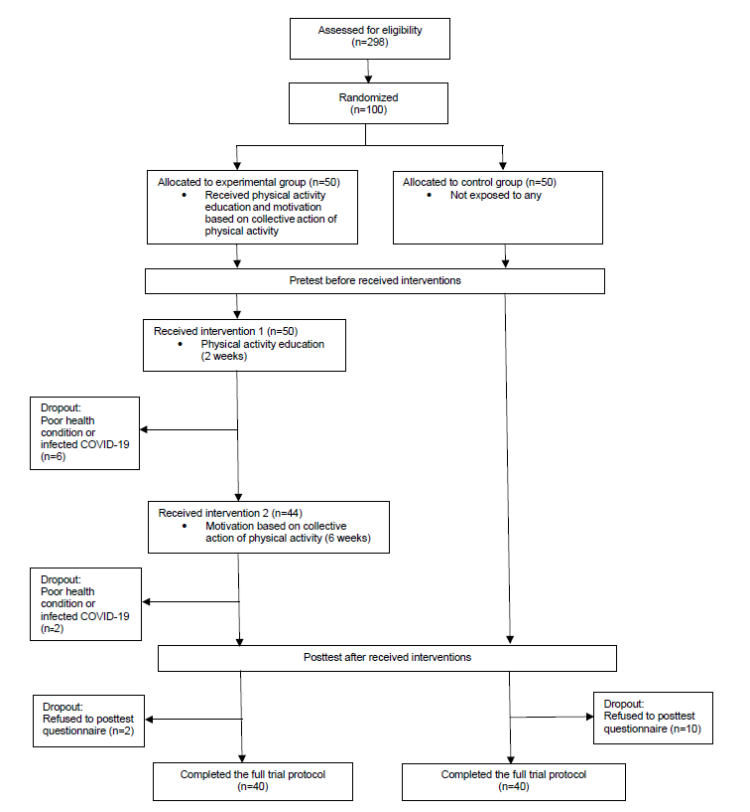
CONSORT (Consolidated Standards of Reporting Trials) flow diagram.

Of the 100 participants, 8 left the study during the trial period and 12 were excluded because they declined to provide posttest data, yielding a 20% (n=20) attrition rate. The remaining 80 (80%) participants in the study comprised 40 individuals each in the experimental and control groups (Figure 2; Multimedia Appendix 1).

### Baseline Characteristics of the Participants

The characteristics and distribution of the sample were described using univariate analysis variables such as percentage, mean, median, and range. The sample of the study comprised Gen Y individuals with similar ages in both experimental (n=40; mean age 29.6, SD 5.52 years) and control (n=40; mean age 29.2, SD 6.48 years) groups. In terms of sex, the proportion of female participants in both intervention and control groups was slightly higher (24/40, 60% and 22/40, 55%, respectively) than male participants (16/40, 40% and 18/40, 45%, respectively). The proportion of participants in the experiment group who were employed as office workers was slightly higher (18/40, 45%) than their counterparts in the control group (14/40, 35%; Table 2). There was no statistically significant difference for general characteristics, health factors, perceptions about PA, and level of moderate to vigorous physical activity (MVPA) at baseline (all *P*>.05). Prior to the intervention, individuals in the experimental group collected a slightly higher average cumulative MVPA (62.15, SD 89.38 minutes per week) than those in the control group (43.55, SD 62.15 minutes per week). Nevertheless, there was no significant difference in the PA level between the 2 groups (*P*=.29; Table 2).

**Table 2:** General characteristics of the sample by group.

Characteristic			Control (n=40)		Experimental (n=40)		*P* value
**Sex, n (%)**							.65^a^
	Male	18 (45)		16 (40)			
	Female	22 (55)		24 (60)			
**Age group (years)** **, n (%)**							.63^a^
	20-29	21 (52)		21 (52)			
	30-39, n (%)	19 (47)		19 (47)			
Age (years), mean (SD)			29.20 (6.48)		29.98 (4.41)		
**Occupation, n (%)**							.33^a^
	Office worker	14 (35)		18 (45)			
	Not an office worker	16 (40)		17 (42)			
	Unemployed	10 (25)		5 (12)			
**Currently have a chronic illness or condition, n (%)**							.80^a^
	Yes	10 (25)		11 (27)			
	No	30 (75)		29 (72)			
**Received education about physical activity, n (%)**							.27^a^
	Yes	29 (72)		31 (77)			
	No	11 (27)		9 (22)			
MVPA^b^ (minutes per week), mean (SD)			43.55 (62.15)		62.15 (89.38)		.29^c^

^a^*P* values are based on the chi-square test

^b^MVPA: moderate to vigorous physical activity.

^c^*P* values are based on the 2-tailed *t* test.

### Comparison in the Stages of Behavioral Change

For bivariate analysis, the significance of association between variables and group-level differences was examined. Before participating in the trial, both sample groups were at the second stage of behavior change (ie, contemplation). According to TTM of behavioral change, contemplation means the individuals intend to change their behavior (into a positive one) within the next 6 months [31]. Upon completing an 8-week intervention, 63% (25/40) of participants in the experimental group and 35% (14/40) participants in the control group improved their level of behavior to the fourth stage (ie, action) by reporting an improvement in their PA for less than 6 months (Table 3). While most of the participants in the experimental group have progressed to the action stage, 50% (20/40) of participants in the control group remained in the contemplation stage. The findings suggest that the intervention helped those in the experimental group to advance in their stage of behavioral readiness, that is, to take action (*χ*^2^_2_=6.1; *P*=.01; Table 3).

**Table 3:** Comparison in the stages of behavioral change of the contemplation stage, preparation stage, and action stage by group and round of data collection. The chi-square test was used^a^.

Round of data collection and group		Stage of change (n=40), n (%)		
		Contemplation	Preparation	Action
**Baseline**				
	Control	40 (100)	0 (0)	0 (0)
	Experimental	40 (100)	0 (0)	0 (0)
**Posttest**				
	Control	20 (50)	6 (15)	14 (35)
	Experimental	6 (15)	9 (22)	25 (62)

^a^Considerations on (1) perceived negative effects of insufficient physical activity (PA), (2) sufficient PA based on the opinions and Global Physical Activity Questionnaire, and (3) intention to increase PA.

### Effectiveness of the Intervention in Increasing Cumulative Minutes of MVPA

To examine the effectiveness of the model, the mean before-and-after values within the same group were compared using the paired 2-tailed *t* test, whereas the means between the 2 groups were compared using the independent samples 2-tailed *t* test. While participants in the control group performed their regular routines, participants in the experimental group were exposed to an 8-week intervention. Health education messages were delivered through selected channels (ie, “Light-hearted” and LINE apps) to provide PA-related knowledge and create awareness of the importance of PA. In the first 2 weeks, various topics were delivered through LINE app and YouTube videos to improve participants’ knowledge in PA, including the importance, cobenefit, and choices of PA, as well as guideline practices for various PA. In the third to sixth week, participants were invited to join Zoom application to collectively set up their goals. Motivational messages and reminders were sent throughout the 4 weeks through the “Light-hearted” app to ensure participants’ compliance.

To test the effectiveness of the intervention, the independent samples 2-tailed *t* test was performed to compare the duration of MVPA (minutes) between participants in the experimental and control groups. After being exposed to the intervention, participants in the intervention group collected a higher cumulative minute of MVPA weekly (283 minutes) than those in the control group (164 minutes), and this was statistically significant (*t*_78_=2.19; *P*=.03; Table 4).

**Table 4:** Differences in the duration of level of MVPA^a^ in minutes per week by group and round of data collection. Independent samples 2-tailed *t* test and paired 2-tailed t test were used.

Variable			MVPA (minutes per week), mean (SD)				Difference (within group), mean (SD)		*t* test (*df*=78)
			Baseline		Posttest				
**Group**									
	Control	43.55 (62.15)		164.05 (202.84)		—^b^		—	
	Experimental	61.75 (89.38)		283.13 (279.22)		221.38 (296.25)		4.73^c^	
Difference (within round of data collection)			—		119.08 (54.57)		—		2.19^d^

^a^MVPA: moderate to vigorous physical activity.

^b^Not available.

^c^Paired 2-tailed *t* test was conducted. Difference indicates a difference of outcome measures between baseline and posttest in each group. Significant difference in comparison with the control group (*P*<.001).

^d^Independent samples 2-tailed *t* test was conducted. Difference indicates the difference of outcome measures between the experimental and control groups after interventions. Significant difference in comparison with the control group (*P*=.03).

The effectiveness of the intervention could also be observed from the increase in the cumulative minutes of MVPA collected by participants in the experimental group. The analysis considered the difference in the duration of PA before and after participating in the study intervention among members of the experimental group using the paired samples 2-tailed *t* test statistic. The analysis found that the mean duration of PA before and after participating in the intervention among the experimental group was significantly different at the *P*<.001 level (*t*_78_=4.73). PA increased by 221.38 minutes, suggesting that the model can significantly increase the PA of Gen Y persons exposed to the intervention (Table 4).

### Effectiveness of the Intervention in Improving the Proportion of Participants With Sufficient MVPA

**Figure 3:**
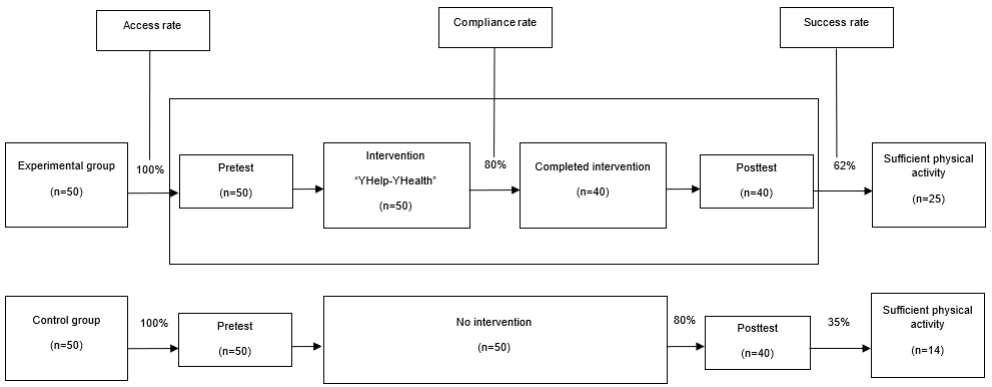
Ability of the intervention to create sufficient physical activity and comparison of behavior change by group and round of data collection sent through the health apps “Light-hearted” and LINE.

In the baseline, 10% (4/40) of participants in the experimental group and 8% (3/40) in the control group met the recommended level of MVPA for adults. After being exposed to the study intervention, the experimental group had more sufficient PA than their counterparts in the control group, and that difference was statistically significant (*P*=.03). Over two-fifths (25/40, 63%) of members in the experimental group had adequate PA compared to only slightly over one-third of the control group (14/40, 35%). With an 80% (80/100) general compliance rate and 63% (25/40) success rate yielding the effect size of the intervention at 28% (effect size=27.5 and number needed to treat=3.64), those who were exposed to the full package of interventions and complied with the intervention (24/25, 96%) recorded positive change and had sufficient PA, and that improvement was statistically significant (*χ*^2^_1_=31.9; *P*<.001). Male adults aged 30-39 years and employed in a nonformal sector are more likely to comply with the intervention compared to female younger adults (20-29 years) who are unemployed or office workers (Figure 3).

## Discussion

### Principal Findings

The effectiveness of a digital group–based activity has been shown to enhance the PA of Gen Y individuals in the intervention group. The COVID-19 pandemic has affected the PA of the population globally. This study found that applying digital group–based activity intervention can help in regenerating PA of Gen Y by considering the context of their lifestyle and habits. The digital group–based activity was able to significantly boost the PA level of behavior of members of the experimental group and shift from the contemplation stage to action. Over 3 in 5 (25/40, 63%) members of the experimental group have successfully changed their behavior by taking action and improving their PA. The findings of this study indicated that the prototype model of digital group–based activity was appropriate for Gen Y as it fits their characteristics and lifestyle. The health information packages in the intervention were beneficial for Gen Y in accelerating their behavior changes, as they contained a series of practical knowledge and examples on PA during COVID-19 pandemic containment periods. The incorporation of the TTM further contributed to the alignment of the intervention with the specific stage of behavioral readiness that varied among individual participants [30,31].

The experimental group showed a greater mean increase in PA than the control group, and that difference is statistically significant. The findings strongly suggest that the prototype of the digital group–based activity to promote PA for Gen Y Thai individuals was successful in building motivation through setting common goals [12,36]. As in-person peer interaction was limited during the containment measures, those Gen Y members who wanted to adopt a healthier lifestyle participated more actively in setting behavior change goals through the digital group–based activity. When engaged with a group, the drive or motivation of each person propels the goals of the group, and that phenomenon can be exploited to increase positive behavior at both the level of behavior and the duration of PA [37].

### Strengths and Limitations

Olson’s collective action theory (1974) [38,39], which focuses on the process of behavior change (ie, motivation) through the creation of collective goals, was applied in this study by leading the participants to believe that a group of their peers had adopted common goals. At least among this sample of Gen Y Thai individuals, this kind of motivation can lead to behavior modification and the ability to achieve the goals as set. The change occurs through direct motivation from intervention activities and indirect motivation from peer group expectations. This process is consistent with collective action theory [38,40], which, according to data in this study, can clearly enhance the PA of Gen Y.

It is noteworthy that members in the control group also reported an increase in PA during the trial period, but not to the same level as those in the experimental group. It is noteworthy that while the control group participants also exhibited an increase in PA during the trial, it was not as significant as the experimental group. This suggests external factors in the societal context may have influenced the behavior of Gen Y participants, such as societal trends emphasizing health and wellness during the pandemic and individual circumstances like remote work or altered routines. In addition, perhaps the mere act of inviting those members of the control group to participate in the study may have been motivational in and of itself [41]. The members of the control group may have become more self-aware of their PA level during the pretest phase and took steps to improve their PA on their own initiative. Or perhaps, the changes in the posttest PA of both groups were due to fewer restriction measures with limited facility opening.

After the completion of the intervention, the experimental group provided valuable feedback, suggesting improvements such as enhancing the frequency of transmission notifications, addressing issues with group chat notifications, and integrating a diverse range of PA selection functions. These suggestions aimed to enhance the overall user experience and make the app more engaging for participants.

All the individuals in the experimental group who dropped out from the study did so because of COVID-19 infection (and were admitted to a clinical facility). Attrition in the control group was due to the inability to participate in the posttest survey. The sample attrition was assumed to be random and, thus, was assumed not to introduce statistical bias in the analysis.

That said, there are certain limitations of this study. For example, the package of interventions was not assessed for efficacy over time after the trial period was complete. This makes it impossible to track the persistence of the recorded PA gains in the intervention group. In addition, this model was designed to be consistent with the Gen Y lifestyle. Even if the intervention improves PA in Gen Y, applying it to other adult populations may have a different effect. In terms of chronic diseases as a variable, the proportion of the sample group who have the disease is not a national proportion, but it is the nature of the sample group who consented to participate and had no effect on the intervention.

### Future Directions

To expand the intervention in other population age groups, it would be necessary to take into account the prevailing stage of behavior and way of life of that population segment. In addition, the findings from this study are short term. Creating profound behavior change to the fourth stage (action) and then on to the fifth stage (maintenance) takes extensive time and a greater range and intensity of interventions than were possible in this study. As a result, there is no guarantee that the observed improvements in the behavior of the experimental group were sustained for any length of time after the end of the trial. However, the fact that the intervention could be delivered on the web means that it would be possible to provide follow-up prompts and ongoing motivation (ie, pandemic or postpandemic) for the target population to sustain or even improve the gains they have made and at very little cost to the program. Future research should compare the collective action of people in a digital community to that of people in a physical or actual community to determine whether or not change occurs. Promoting PA in the digital community can be a very cost-effective way to increase the likelihood that younger individuals will become more physically active. Since the digital community can be implemented on numerous internet platforms, its theoretical reach is essentially limitless. The digital community can also connect individuals to comparable groups locally and globally. Finally, the digital community is substantially less expensive to implement than a comparable physical community intervention.

### Conclusions

There was a significant change in the PA level of participants in the intervention group who were exposed to digital group–based activity for 8 weeks. The model was effective in promoting PA for members of Gen Y seeking to change their health behavior through the setting of common goals and the provision of adequate health-related information. This study showed that the prototype model was tailored to the prevailing lifestyle of Gen Y and, thus, could be integrated in their daily routine with least disruption.

## Appendix

Multimedia Appendix 1 CONSORT-eHEALTH checklist (V 1.6.1).

## Abbreviations

Gen Y: Generation Y
MVPA: moderate to vigorous physical activity
PA: physical activity
TTM: transtheoretical model
WHO: World Health Organization
