# Supplementary material for: Digital Group–Based Intervention for Physical Activity Promotion Among Thai Adults During the COVID-19 Lockdown: Randomized Controlled Trial
Source: J Med Internet Res. 2024 Jan 31;26:e43366. doi: 10.2196/43366 (PMC10867743; doi:10.2196/43366)
Supplement: Multimedia Appendix 1 [file jmir_v26i1e43366_app1.pdf]

CONSORT-EHEALTH (V 1.6.1) - Submission/Publication Form

The CONSORT-EHEALTH checklist is intended for authors of randomized trials evaluating web-based and Internet-based applications/interventions, including mobile interventions, electronic games (incl multiplayer games), social media, certain telehealth applications, and other interactive and/or networked electronic applications. Some of the items (e.g. all subitems under item 5 - description of the intervention) may also be applicable for other study designs.

The goal of the CONSORT EHEALTH checklist and guideline is to be
a) a guide for reporting for authors of RCTs,
b) to form a basis for appraisal of an ehealth trial (in terms of validity)

CONSORT-EHEALTH items/subitems are MANDATORY reporting items for studies published in the Journal of Medical Internet Research and other journals / scientific societies endorsing the checklist.

Items numbered 1., 2., 3., 4a., 4b etc are original CONSORT or CONSORT-NPT (non-pharmacologic treatment) items.
Items with Roman numerals (i., ii, iii, iv etc.) are CONSORT-EHEALTH extensions/clarifications.

As the CONSORT-EHEALTH checklist is still considered in a formative stage, we would ask that you also RATE ON A SCALE OF 1-5 how important/useful you feel each item is FOR THE PURPOSE OF THE CHECKLIST and reporting guideline (optional).

Mandatory reporting items are marked with a red *.
In the textboxes, either copy & paste the relevant sections from your manuscript into this form - please include any quotes from your manuscript in QUOTATION MARKS,
or answer directly by providing additional information not in the manuscript, or elaborating on why the item was not relevant for this study.

YOUR ANSWERS WILL BE PUBLISHED AS A SUPPLEMENTARY FILE TO YOUR PUBLICATION IN JMIR AND ARE CONSIDERED PART OF YOUR PUBLICATION (IF ACCEPTED).
Please fill in these questions diligently. Information will not be copyedited, so please use proper spelling and grammar, use correct capitalization, and avoid abbreviations.

DO NOT FORGET TO SAVE AS PDF _AND_ CLICK THE SUBMIT BUTTON SO YOUR ANSWERS ARE IN OUR DATABASE !!!

Citation Suggestion (if you append the pdf as Appendix we suggest to cite this paper in the caption):
Eysenbach G, CONSORT-EHEALTH Group

CONSORT-EHEALTH: Improving and Standardizing Evaluation Reports of Web-based and Mobile Health Interventions
J Med Internet Res 2011;13(4):e126
URL: http://www.jmir.org/2011/4/e126/
doi: 10.2196/jmir.1923
PMID: 22209829

p.nanthawan1412@gmail.com สลับบัญชี

ไม่ใช้ร่วมกัน

บันทึกฉบับร่างแล้ว

* ระบุว่าเป็นคำถามที่จำเป็น

Your name *

First Last

Piyawat Katewongsa

Primary Affiliation (short), City, Country *

University of Toronto, Toronto, Canada

Institute for Population and Social Research, M

Your e-mail address *

abc@gmail.com

piyawat.kat@mahidol.edu

Title of your manuscript *

Provide the (draft) title of your manuscript.

Online group-based intervention for physical activity promotion among Thai adults during the COVID-19 lockdown: A Randomized Controlled Trial

Name of your App/Software/Intervention *

If there is a short and a long/alternate name, write the short name first and add the long name in brackets.

health application “Light-hearted”, and interver

Evaluated Version (if any)

e.g. "V1", "Release 2017-03-01", "Version 2.0.27913"

Version 1

Language(s) *

What language is the intervention/app in? If multiple languages are available, separate by comma (e.g. "English, French")

Thai language

URL of your Intervention Website or App

e.g. a direct link to the mobile app on app in appstore (itunes, Google Play), or URL of the website. If the intervention is a DVD or hardware, you can also link to an Amazon page.

https://play.google.com/store/apps/details?id=co.popmed.baojai

URL of an image/screenshot (optional)

คำตอบของคุณ

Accessibility *

Can an enduser access the intervention presently?

- ○ access is free and open
- ○ access only for special usergroups, not open
- ◉ access is open to everyone, but requires payment/subscription/in-app purchases
- ○ app/intervention no longer accessible
- ○ อื่นๆ:

Primary Medical Indication/Disease/Condition *

e.g. "Stress", "Diabetes", or define the target group in brackets after the condition, e.g. "Autism (Parents of children with)", "Alzheimers (Informal Caregivers of)"

Having insufficient Physical Activity as recomr

Primary Outcomes measured in trial *

comma-separated list of primary outcomes reported in the trial

Effectiveness of the model promote physical a

Secondary/other outcomes

Are there any other outcomes the intervention is expected to affect?

Sufficient physical activity

Recommended "Dose" *

What do the instructions for users say on how often the app should be used?

- (●) Approximately Daily
- ( ) Approximately Weekly
- ( ) Approximately Monthly
- ( ) Approximately Yearly
- ( ) "as needed"
- ( ) อื่นๆ: ____

Approx. Percentage of Users (starters) still using the app as recommended after 3 months *

- [x] unknown / not evaluated
- [ ] 0-10%
- [ ] 11-20%
- [ ] 21-30%
- [ ] 31-40%
- [ ] 41-50%
- [ ] 51-60%
- [ ] 61-70%
- [ ] 71%-80%
- [ ] 81-90%
- [ ] 91-100%
- [ ] อื่นๆ:

Overall, was the app/intervention effective? *

- [x] yes: all primary outcomes were significantly better in intervention group vs control
- [ ] partly: SOME primary outcomes were significantly better in intervention group vs control
- [ ] no statistically significant difference between control and intervention
- [ ] potentially harmful: control was significantly better than intervention in one or more outcomes
- [ ] inconclusive: more research is needed
- [ ] อื่นๆ:

Article Preparation Status/Stage *

At which stage in your article preparation are you currently (at the time you fill in this form)

- ○ not submitted yet - in early draft status
- ○ not submitted yet - in late draft status, just before submission
- ○ submitted to a journal but not reviewed yet
- ◉ submitted to a journal and after receiving initial reviewer comments
- ○ submitted to a journal and accepted, but not published yet
- ○ published
- ○ อื่นๆ: ______

Journal *

If you already know where you will submit this paper (or if it is already submitted), please provide the journal name (if it is not JMIR, provide the journal name under "other")

- ○ not submitted yet / unclear where I will submit this
- ◉ Journal of Medical Internet Research (JMIR)
- ○ JMIR mHealth and UHealth
- ○ JMIR Serious Games
- ○ JMIR Mental Health
- ○ JMIR Public Health
- ○ JMIR Formative Research
- ○ Other JMIR sister journal
- ○ อื่นๆ: ______

Is this a full powered effectiveness trial or a pilot/feasibility trial? *

- ○ Pilot/feasibility
- ◉ Fully powered

Manuscript tracking number *

If this is a JMIR submission, please provide the manuscript tracking number under "other" (The ms tracking number can be found in the submission acknowledgement email, or when you login as author in JMIR. If the paper is already published in JMIR, then the ms tracking number is the four-digit number at the end of the DOI, to be found at the bottom of each published article in JMIR)

- ○ no ms number (yet) / not (yet) submitted to / published in JMIR
- ◉ อื่นๆ: 43366

TITLE AND ABSTRACT

1a) TITLE: Identification as a randomized trial in the title

1a) Does your paper address CONSORT item 1a? *

I.e does the title contain the phrase "Randomized Controlled Trial"? (if not, explain the reason under "other")

- ◉ yes
- ○ อื่นๆ:

1a-i) Identify the mode of delivery in the title

Identify the mode of delivery. Preferably use “web-based” and/or “mobile” and/or “electronic game” in the title. Avoid ambiguous terms like “online”, “virtual”, “interactive”. Use “Internet-based” only if Intervention includes non-web-based Internet components (e.g. email), use “computer-based” or “electronic” only if offline products are used. Use “virtual” only in the context of “virtual reality” (3-D worlds). Use “online” only in the context of “online support groups”. Complement or substitute product names with broader terms for the class of products (such as “mobile” or “smart phone” instead of “iphone”), especially if the application runs on different platforms.

subitem not at all important

1 ○

2 ○

3 ○

4 ○

5 ◉

essential

ล้างสิ่งที่เลือก

Does your paper address subitem 1a-i? *

Copy and paste relevant sections from manuscript title (include quotes in quotation marks "like this" to indicate direct quotes from your manuscript), or elaborate on this item by providing additional information not in the ms, or briefly explain why the item is not applicable/relevant for your study

"Online group-based intervention" for physical activity promotion among Thai adults during the COVID-19 lockdown: A Randomized Controlled Trial

1a-ii) Non-web-based components or important co-interventions in title

Mention non-web-based components or important co-interventions in title, if any (e.g., “with telephone support”).

subitem not at all important

- 1 (selected)
- 2
- 3
- 4
- 5

essential

ล้างสิ่งที่เลือก

Does your paper address subitem 1a-ii?

Copy and paste relevant sections from manuscript title (include quotes in quotation marks "like this" to indicate direct quotes from your manuscript), or elaborate on this item by providing additional information not in the ms, or briefly explain why the item is not applicable/relevant for your study

คำตอบของคุณ

1a-iii) Primary condition or target group in the title

Mention primary condition or target group in the title, if any (e.g., “for children with Type I Diabetes”) Example: A Web-based and Mobile Intervention with Telephone Support for Children with Type I Diabetes: Randomized Controlled Trial

subitem not at all important

1 ○

2 ○

3 ○

4 ○

5 ◉

essential

ล้างสิ่งที่เลือก

Does your paper address subitem 1a-iii? *

Copy and paste relevant sections from manuscript title (include quotes in quotation marks "like this" to indicate direct quotes from your manuscript), or elaborate on this item by providing additional information not in the ms, or briefly explain why the item is not applicable/relevant for your study

Online group-based intervention for physical activity promotion among "Thai adults" during the COVID-19 lockdown: A Randomized Controlled Trial

1b) ABSTRACT: Structured summary of trial design, methods, results, and conclusions

NPT extension: Description of experimental treatment, comparator, care providers, centers, and blinding status.

1b-i) Key features/functionalities/components of the intervention and comparator in the METHODS section of the ABSTRACT

Mention key features/functionalities/components of the intervention and comparator in the abstract. If possible, also mention theories and principles used for designing the site. Keep in mind the needs of systematic reviewers and indexers by including important synonyms. (Note: Only report in the abstract what the main paper is reporting. If this information is missing from the main body of text, consider adding it)

subitem not at all important

1 ○

2 ○

3 ○

4 ○

5 ◉

essential

ล้างสิ่งที่เลือก

Does your paper address subitem 1b-i? *

Copy and paste relevant sections from the manuscript abstract (include quotes in quotation marks "like this" to indicate direct quotes from your manuscript), or elaborate on this item by providing additional information not in the ms, or briefly explain why the item is not applicable/relevant for your study

This was a parallel two-arm randomized controlled trial, with single-blind allocation to experimental and control groups, and pre- and post-test measurements. The sample comprised of 100 Gen Y individuals who met the inclusion criteria. Both groups were matched for background characteristics. "The two, 8-week intervention activities were (a) 2 weeks of education; and (b) 6 weeks of motivation."

1b-ii) Level of human involvement in the METHODS section of the ABSTRACT

Clarify the level of human involvement in the abstract, e.g., use phrases like “fully automated” vs. “therapist/nurse/care provider/physician-assisted” (mention number and expertise of providers involved, if any). (Note: Only report in the abstract what the main paper is reporting. If this information is missing from the main body of text, consider adding it)

subitem not at all important

1 ◉

2 ○

3 ○

4 ○

5 ○

essential

ล้างสิ่งที่เลือก

Does your paper address subitem 1b-ii?

Copy and paste relevant sections from the manuscript abstract (include quotes in quotation marks "like this" to indicate direct quotes from your manuscript), or elaborate on this item by providing additional information not in the ms, or briefly explain why the item is not applicable/relevant for your study

คำตอบของคุณ

1b-iii) Open vs. closed, web-based (self-assessment) vs. face-to-face assessments in the METHODS section of the ABSTRACT

Mention how participants were recruited (online vs. offline), e.g., from an open access website or from a clinic or a closed online user group (closed usergroup trial), and clarify if this was a purely web-based trial, or there were face-to-face components (as part of the intervention or for assessment). Clearly say if outcomes were self-assessed through questionnaires (as common in web-based trials). Note: In traditional offline trials, an open trial (open-label trial) is a type of clinical trial in which both the researchers and participants know which treatment is being administered. To avoid confusion, use “blinded” or “unblinded” to indicated the level of blinding instead of “open”, as “open” in web-based trials usually refers to “open access” (i.e. participants can self-enrol). (Note: Only report in the abstract what the main paper is reporting. If this information is missing from the main body of text, consider adding it)

subitem not at all important

1 ○

2 ○

3 ◉

4 ○

5 ○

essential

ล้างสิ่งที่เลือก

Does your paper address subitem 1b-iii?

Copy and paste relevant sections from the manuscript abstract (include quotes in quotation marks "like this" to indicate direct quotes from your manuscript), or elaborate on this item by providing additional information not in the ms, or briefly explain why the item is not applicable/relevant for your study

"using Zoom meetings application"

1b-iv) RESULTS section in abstract must contain use data

Report number of participants enrolled/assessed in each group, the use/uptake of the intervention (e.g., attrition/adherence metrics, use over time, number of logins etc.), in addition to primary/secondary outcomes. (Note: Only report in the abstract what the main paper is reporting. If this information is missing from the main body of text, consider adding it)

subitem not at all important

1 ○

2 ○

3 ○

4 ○

5 ◉

essential

ล้างสิ่งที่เลือก

Does your paper address subitem 1b-iv?

Copy and paste relevant sections from the manuscript abstract (include quotes in quotation marks "like this" to indicate direct quotes from your manuscript), or elaborate on this item by providing additional information not in the ms, or briefly explain why the item is not applicable/relevant for your study

คำตอบของคุณ

1b-v) CONCLUSIONS/DISCUSSION in abstract for negative trials

Conclusions/Discussions in abstract for negative trials: Discuss the primary outcome - if the trial is negative (primary outcome not changed), and the intervention was not used, discuss whether negative results are attributable to lack of uptake and discuss reasons. (Note: Only report in the abstract what the main paper is reporting. If this information is missing from the main body of text, consider adding it)

subitem not at all important

- 1 ◉
- 2 ○
- 3 ○
- 4 ○
- 5 ○

essential

ล้างสิ่งที่เลือก

Does your paper address subitem 1b-v?

Copy and paste relevant sections from the manuscript abstract (include quotes in quotation marks "like this" to indicate direct quotes from your manuscript), or elaborate on this item by providing additional information not in the ms, or briefly explain why the item is not applicable/relevant for your study

คำตอบของคุณ

INTRODUCTION

2a) In INTRODUCTION: Scientific background and explanation of rationale

2a-i) Problem and the type of system/solution

Describe the problem and the type of system/solution that is object of the study: intended as stand-alone intervention vs. incorporated in broader health care program? Intended for a particular patient population? Goals of the intervention, e.g., being more cost-effective to other interventions, replace or complement other solutions? (Note: Details about the intervention are provided in “Methods” under 5)

subitem not at all important

1 ○

2 ○

3 ○

4 ○

5 ◉

essential

ล้างสิ่งที่เลือก

Does your paper address subitem 2a-i? *

Copy and paste relevant sections from the manuscript (include quotes in quotation marks "like this" to indicate direct quotes from your manuscript), or elaborate on this item by providing additional information not in the ms, or briefly explain why the item is not applicable/relevant for your study

of premature deaths due to Non-Communicable Disease (NCDs), lower incident of hypertension, type-2 diabetes, and cancer; improved cognitive function, and better mental health outcomes."
"The COVID-19 pandemic has forced nationwide lockdowns and most of the countries to use social distancing measures and policies to encourage people to stay at home to reduce the spread of disease. These measures have restricted people's movement and lead to reduction in physical activity globally. With limited opportunity to engage in regular outdoors physical activity during containment periods, lifestyle changes are required."

2a-ii) Scientific background, rationale: What is known about the (type of) system

Scientific background, rationale: What is known about the (type of) system that is the object of the study (be sure to discuss the use of similar systems for other conditions/diagnoses, if appropiate), motivation for the study, i.e. what are the reasons for and what is the context for this specific study, from which stakeholder viewpoint is the study performed, potential impact of findings [2]. Briefly justify the choice of the comparator.

subitem not at all important

1 ○

2 ○

3 ○

4 ○

5 ◉

essential

ล้างสิ่งที่เลือก

Does your paper address subitem 2a-ii? *

Copy and paste relevant sections from the manuscript (include quotes in quotation marks "like this" to indicate direct quotes from your manuscript), or elaborate on this item by providing additional information not in the ms, or briefly explain why the item is not applicable/relevant for your study

"The importance of physical activity (PA) for health has been documented worldwide. For adults, regular and adequate physical activity has been associated with lower risk of premature deaths due to Non-Communicable Disease (NCDs), lower incident of hypertension, type-2 diabetes, and cancer; improved cognitive function, and better mental health outcomes. Nevertheless, although these benefits have been acknowledged and public health promotion efforts have been undertaken, physical inactivity remains a global public health problem."
"The COVID-19 pandemic has forced nationwide lockdowns and most of the countries to use social distancing measures and policies to encourage people to stay at home to reduce the spread of disease. These measures have restricted people's movement and lead to reduction in PA globally". With limited opportunity to engage in regular outdoors PA during containment periods, lifestyle changes are required. Health promotion strategies have then focused the message to promote home-based PA and encourage population at all ages to stay active while at home. WHO initiated with 'Healthy at Home' campaign and was followed by many countries with similar focus: encouraging home-based PA to regenerate PA of the population."
"Home-based PA was promoted using various channels, especially online media and online group-based activity which provided recommendation of home-based exercise, sample videos of various training programs, free virtual exercise classes on the web or on electronic platforms such as YouTube, Facebook, Twitter or via an app or online. A few studies have documented the positive effect of home-based PA promotion (e.g., Fit from Home (FFH) in improving PA of the population."
"While the actual community interaction was limited during the pandemic, online community has been increasingly demanded and becoming an everyday part of every individual's life. This community is a place for people to gather online, who may or may not meet one another face to face, to stay in touch with or perform certain activities in the groups they belong to. The virtual engagement creates and maintains relationships between group members in the online group-based activity, affecting both psychological and social motivation, and this can lead to behavior modification of members within the group.
With more than half of the people globally now use social community, Online group-based activity (a virtual community platform) has the potential to promote PA during the pandemic. Unlike the mass campaigns that are addressed to general population, information delivered in the online group-based activity targeted specific group who share similar interest. Good engagement within the group increases the effectiveness of the messages in driving the behavior changes of its member."

2b) In INTRODUCTION: Specific objectives or hypotheses

Does your paper address CONSORT subitem 2b? *

Copy and paste relevant sections from the manuscript (include quotes in quotation marks "like this" to indicate direct quotes from your manuscript), or elaborate on this item by providing additional information not in the ms, or briefly explain why the item is not applicable/relevant for your study

"This research aimed to examine the effectiveness of an online group-based activity in promoting physical activity (PA) among Gen Y population in Thailand."

METHODS

3a) Description of trial design (such as parallel, factorial) including allocation ratio

Does your paper address CONSORT subitem 3a? *

Copy and paste relevant sections from the manuscript (include quotes in quotation marks "like this" to indicate direct quotes from your manuscript), or elaborate on this item by providing additional information not in the ms, or briefly explain why the item is not applicable/relevant for your study

"A parallel two-arm randomized controlled trial (RCT) design, with single-blind assignment of subjects to experimental and control groups was employed. Outcomes were measured by pre- and post-test assessments (pretest-posttest design with control group). Matching between the experimental and control group was performed by aligning the individual characteristics, e.g., sex, age group, and primary occupation by using Probability Random Sampling. While the intervention group received a set of interventions, the control group performed their routines without any intervention."
"The population universe in this research is Thais adults who belong to the gen Y and who have access to the Internet, or a total of 17,644,290 people. The Gen Y population was defined as a group of the people those were born between 1981 and 2000. This segment of the population is critical for the future development of the country, both in the labor market and in terms of the economy (insofar as the Gen Y group dominates the spending power of the country). This population was randomly sampled (with probability known) to provide information on Thailand Physical Activity Surveillance System. The prescribed sample size was calculated by considering the value alpha = 0.05, effect size = 0.5 (a medium effect size from the previous study), power = 0.95, and a 2-tail test. To reduce attrition due to withdrawal from the study or refusal to participate, the sample size was increased from the minimum prescribed number (54 persons), resulting in a total sample of 100 people, evenly divided into experimental and

3b) Important changes to methods after trial commencement (such as eligibility criteria), with reasons

Does your paper address CONSORT subitem 3b? *

Copy and paste relevant sections from the manuscript (include quotes in quotation marks "like this" to indicate direct quotes from your manuscript), or elaborate on this item by providing additional information not in the ms, or briefly explain why the item is not applicable/relevant for your study

"Methods are not changed after trial because we can monitor the project's progress."

3b-i) Bug fixes, Downtimes, Content Changes

Bug fixes, Downtimes, Content Changes: ehealth systems are often dynamic systems. A description of changes to methods therefore also includes important changes made on the intervention or comparator during the trial (e.g., major bug fixes or changes in the functionality or content) (5-iii) and other “unexpected events” that may have influenced study design such as staff changes, system failures/downtimes, etc. [2].

subitem not at all important

1 (selected)

2

3

4

5

essential

ล้างสิ่งที่เลือก

Does your paper address subitem 3b-i?

Copy and paste relevant sections from the manuscript (include quotes in quotation marks "like this" to indicate direct quotes from your manuscript), or elaborate on this item by providing additional information not in the ms, or briefly explain why the item is not applicable/relevant for your study

คำตอบของคุณ

4a) Eligibility criteria for participants

Does your paper address CONSORT subitem 4a? *

Copy and paste relevant sections from the manuscript (include quotes in quotation marks "like this" to indicate direct quotes from your manuscript), or elaborate on this item by providing additional information not in the ms, or briefly explain why the item is not applicable/relevant for your study

"Eligible participants were those who met the inclusion criteria of having insufficient physical activity as recommended by WHO, and were in the second stage of behavior change (i.e., contemplation) because 1 of 3 Gen Y are in this stage, and who had access to the Internet at the time of the survey. Simple random sampling with replacement was used to randomly assign the participants into experimental group or control group. In case of sample rejection, a suitable replacement was selected."

4a-i) Computer / Internet literacy

Computer / Internet literacy is often an implicit “de facto” eligibility criterion - this should be explicitly clarified.

subitem not at all important

- 1 (selected)
- 2
- 3
- 4
- 5

essential

ล้างสิ่งที่เลือก

Does your paper address subitem 4a-i?

Copy and paste relevant sections from the manuscript (include quotes in quotation marks "like this" to indicate direct quotes from your manuscript), or elaborate on this item by providing additional information not in the ms, or briefly explain why the item is not applicable/relevant for your study

คำตอบของคุณ

4a-ii) Open vs. closed, web-based vs. face-to-face assessments:

Open vs. closed, web-based vs. face-to-face assessments: Mention how participants were recruited (online vs. offline), e.g., from an open access website or from a clinic, and clarify if this was a purely web-based trial, or there were face-to-face components (as part of the intervention or for assessment), i.e., to what degree got the study team to know the participant. In online-only trials, clarify if participants were quasi-anonymous and whether having multiple identities was possible or whether technical or logistical measures (e.g., cookies, email confirmation, phone calls) were used to detect/prevent these.

subitem not at all important

1 ○

2 ○

3 ○

4 ○

5 ◉

essential

ล้างสิ่งที่เลือก

Does your paper address subitem 4a-ii? *

Copy and paste relevant sections from the manuscript (include quotes in quotation marks "like this" to indicate direct quotes from your manuscript), or elaborate on this item by providing additional information not in the ms, or briefly explain why the item is not applicable/relevant for your study

"This online population was randomly sampled (with probability known) to provide information on Thailand Physical Activity Surveillance System"

4a-iii) Information giving during recruitment

Information given during recruitment. Specify how participants were briefed for recruitment and in the informed consent procedures (e.g., publish the informed consent documentation as appendix, see also item X26), as this information may have an effect on user self-selection, user expectation and may also bias results.

subitem not at all important

1 ○

2 ○

3 ○

4 ○

5 ◉

essential

ล้างสิ่งที่เลือก

Does your paper address subitem 4a-iii?

Copy and paste relevant sections from the manuscript (include quotes in quotation marks "like this" to indicate direct quotes from your manuscript), or elaborate on this item by providing additional information not in the ms, or briefly explain why the item is not applicable/relevant for your study

"After the sampling process completed, the consent and voluntary participation were inquired. In case of sample rejection, the new random sample with the same eligible and method was replaced."
"All participants agreed in advance to provide data to the researchers and were assured that all their information would be kept confidential. Participants provided their consent to be included in the study by click on the agreement box available in the Lime-survey web application. No personal information of the participant was collected or used in a way that would identify the data provider. If the intervention was successful in the experimental group, the intervention would also be offered to the control group participants."

4b) Settings and locations where the data were collected

Does your paper address CONSORT subitem 4b? *

Copy and paste relevant sections from the manuscript (include quotes in quotation marks "like this" to indicate direct quotes from your manuscript), or elaborate on this item by providing additional information not in the ms, or briefly explain why the item is not applicable/relevant for your study

"For the prototype model in this study, the intervention covered 8 weeks of activity (from August 22 to October 16, 2021). The designed interventions consisted of education and motivation (as per theory), with the intervention activities implemented one by one at specified intervals, delivered through online platforms (health apps, Line official account)."

4b-i) Report if outcomes were (self-)assessed through online questionnaires

Clearly report if outcomes were (self-)assessed through online questionnaires (as common in web-based trials) or otherwise.

subitem not at all important

1 ○

2 ○

3 ○

4 ○

5 ◉

essential

ล้างสิ่งที่เลือก

Does your paper address subitem 4b-i? *

Copy and paste relevant sections from the manuscript (include quotes in quotation marks "like this" to indicate direct quotes from your manuscript), or elaborate on this item by providing additional information not in the ms, or briefly explain why the item is not applicable/relevant for your study

"A structured questionnaire was used as the data collection tool. Physical activity and sedentary behavior was assessed by using the Global Physical Activity Questionnaire (GPAQ) version 2, while behavior assessment employed the standardized and validated Trans-Theoretical Model (TTM) questionnaire. The questions were administered online in the Limesurvey web application and was designed appropriately for the respondents to fill by themselves i.e., Physical Activity for example."

4b-ii) Report how institutional affiliations are displayed

Report how institutional affiliations are displayed to potential participants [on ehealth media], as affiliations with prestigious hospitals or universities may affect volunteer rates, use, and reactions with regards to an intervention.(Not a required item – describe only if this may bias results)

subitem not at all important

1 (selected)

2

3

4

5

essential

ล้างสิ่งที่เลือก

Does your paper address subitem 4b-ii?

Copy and paste relevant sections from the manuscript (include quotes in quotation marks "like this" to indicate direct quotes from your manuscript), or elaborate on this item by providing additional information not in the ms, or briefly explain why the item is not applicable/relevant for your study

คำตอบของคุณ

5) The interventions for each group with sufficient details to allow replication, including how and when they were actually administered

5-i) Mention names, credential, affiliations of the developers, sponsors, and owners

Mention names, credential, affiliations of the developers, sponsors, and owners [6] (if authors/evaluators are owners or developer of the software, this needs to be declared in a “Conflict of interest” section or mentioned elsewhere in the manuscript).

subitem not at all important

1 ○

2 ○

3 ○

4 ◉

5 ○

essential

ล้างสิ่งที่เลือก

Does your paper address subitem 5-i?

Copy and paste relevant sections from the manuscript (include quotes in quotation marks "like this" to indicate direct quotes from your manuscript), or elaborate on this item by providing additional information not in the ms, or briefly explain why the item is not applicable/relevant for your study

"support from the Biomed lab, the health application “Light-hearted” from Popmed Co. Ltd., and the Department of Biomedical Engineering, Faculty of Engineering, Mahidol University, Thailand."

5-ii) Describe the history/development process

Describe the history/development process of the application and previous formative evaluations (e.g., focus groups, usability testing), as these will have an impact on adoption/use rates and help with interpreting results.

subitem not at all important

1 ◉

2 ○

3 ○

4 ○

5 ○

essential

ล้างสิ่งที่เลือก

Does your paper address subitem 5-ii?

Copy and paste relevant sections from the manuscript (include quotes in quotation marks "like this" to indicate direct quotes from your manuscript), or elaborate on this item by providing additional information not in the ms, or briefly explain why the item is not applicable/relevant for your study

คำตอบของคุณ

5-iii) Revisions and updating

Revisions and updating. Clearly mention the date and/or version number of the application/intervention (and comparator, if applicable) evaluated, or describe whether the intervention underwent major changes during the evaluation process, or whether the development and/or content was “frozen” during the trial. Describe dynamic components such as news feeds or changing content which may have an impact on the replicability of the intervention (for unexpected events see item 3b).

subitem not at all important

- 1 (selected)
- 2
- 3
- 4
- 5

essential

ล้างสิ่งที่เลือก

Does your paper address subitem 5-iii?

Copy and paste relevant sections from the manuscript (include quotes in quotation marks "like this" to indicate direct quotes from your manuscript), or elaborate on this item by providing additional information not in the ms, or briefly explain why the item is not applicable/relevant for your study

คำตอบของคุณ

5-iv) Quality assurance methods

Provide information on quality assurance methods to ensure accuracy and quality of information provided [1], if applicable.

subitem not at all important

1 ◉

2 ○

3 ○

4 ○

5 ○

essential

ล้างสิ่งที่เลือก

Does your paper address subitem 5-iv?

Copy and paste relevant sections from the manuscript (include quotes in quotation marks "like this" to indicate direct quotes from your manuscript), or elaborate on this item by providing additional information not in the ms, or briefly explain why the item is not applicable/relevant for your study

คำตอบของคุณ

5-v) Ensure replicability by publishing the source code, and/or providing screenshots/screen-capture video, and/or providing flowcharts of the algorithms used

Ensure replicability by publishing the source code, and/or providing screenshots/screen-capture video, and/or providing flowcharts of the algorithms used. Replicability (i.e., other researchers should in principle be able to replicate the study) is a hallmark of scientific reporting.

subitem not at all important

1 (selected)

2

3

4

5

essential

ล้างสิ่งที่เลือก

Does your paper address subitem 5-v?

Copy and paste relevant sections from the manuscript (include quotes in quotation marks "like this" to indicate direct quotes from your manuscript), or elaborate on this item by providing additional information not in the ms, or briefly explain why the item is not applicable/relevant for your study

คำตอบของคุณ

5-vi) Digital preservation

Digital preservation: Provide the URL of the application, but as the intervention is likely to change or disappear over the course of the years; also make sure the intervention is archived (Internet Archive, webcitation.org, and/or publishing the source code or screenshots/videos alongside the article). As pages behind login screens cannot be archived, consider creating demo pages which are accessible without login.

subitem not at all important

1 (●)

2 ( )

3 ( )

4 ( )

5 ( )

essential

ล้างสิ่งที่เลือก

Does your paper address subitem 5-vi?

Copy and paste relevant sections from the manuscript (include quotes in quotation marks "like this" to indicate direct quotes from your manuscript), or elaborate on this item by providing additional information not in the ms, or briefly explain why the item is not applicable/relevant for your study

คำตอบของคุณ

5-vii) Access

Access: Describe how participants accessed the application, in what setting/context, if they had to pay (or were paid) or not, whether they had to be a member of specific group. If known, describe how participants obtained “access to the platform and Internet” [1]. To ensure access for editors/reviewers/readers, consider to provide a “backdoor” login account or demo mode for reviewers/readers to explore the application (also important for archiving purposes, see vi).

subitem not at all important

1 (selected)

2

3

4

5

essential

ล้างสิ่งที่เลือก

Does your paper address subitem 5-vii? *

Copy and paste relevant sections from the manuscript (include quotes in quotation marks "like this" to indicate direct quotes from your manuscript), or elaborate on this item by providing additional information not in the ms, or briefly explain why the item is not applicable/relevant for your study

"The health application Light-hearted, and LINE was installed after the intervention group has consented to participate in the intervention."

5-viii) Mode of delivery, features/functionalities/components of the intervention and comparator, and the theoretical framework

Describe mode of delivery, features/functionalities/components of the intervention and comparator, and the theoretical framework [6] used to design them (instructional strategy [1], behaviour change techniques, persuasive features, etc., see e.g., [7, 8] for terminology). This includes an in-depth description of the content (including where it is coming from and who developed it) [1],” whether [and how] it is tailored to individual circumstances and allows users to track their progress and receive feedback” [6]. This also includes a description of communication delivery channels and – if computer-mediated communication is a component – whether communication was synchronous or asynchronous [6]. It also includes information on presentation strategies [1], including page design principles, average amount of text on pages, presence of hyperlinks to other resources, etc. [1].

subitem not at all important

1 ○

2 ○

3 ○

4 ○

5 ◉

essential

ล้างสิ่งที่เลือก

Does your paper address subitem 5-viii? *

Copy and paste relevant sections from the manuscript (include quotes in quotation marks "like this" to indicate direct quotes from your manuscript), or elaborate on this item by providing additional information not in the ms, or briefly explain why the item is not applicable/relevant for your study

"The Light-hearted was designed as a mobile-based application considering Gen Y population were the heavy users of digital media. This application recorded the number of steps and other PA including biking, swimming, walking, boxing, volleyball, badminton, etc. Participants were also requested to record their daily calorie intake. Additional features including health information and feedback mechanism (i.e., suggestion box) were also made available to improve participants engagement apart from chat box."
"For the prototype model in this study, the intervention covered 8 weeks of activity (from August 22 to October 16, 2021). The designed interventions consisted of education and motivation (as per theory), with the intervention activities implemented one by one at specified intervals, delivered through online platforms (health apps, Line official account)."
"Providing knowledge to increase awareness.
This part of the intervention lasted 2 weeks. This activity aimed to build knowledge and understanding of guidelines on PA for the intervention group. In the first week, participants were exposed to health education messages (infographic and video clips) sent through a health application "Light-hearted," and LINE. The messages covered PA self-assessment, the importance of PA, advantages, and disadvantages of the original behavior, as well as co-benefits of sufficient PA, and dramatic relief. In the second week, the health education message described the practice of various physical activities. This included information about alternatives to PA, and PA for the working-age population, both in the home/around the house, at work, and in public areas. The intention was to add a variety of options for PA. The video was produced by Social Marketing Team of XXXXXX, was in a public domain (XXXXXX YouTube channel) and free to be used. The videos contained messages on the causes of NCDs, shared experience and motivation for regular exercise."
"Creating Motivation.
A total of 6 weeks was spent to apply the Collective Action Theory, which was the main process hypothesized to drive behavior change in this study. Target groups set goals for PA together, with a time limit and group consensus as to when the goal was to be achieved. The Collective Action Theory prescribes both internal and external motivation. Both types of motivation were directly targeted by the intervention activity, and indirectly targeted through discussion and encouragement via group chat as an additional impetus to behavior change. The process started with setting group goals by using Zoom meetings to discuss PA goals in the entire experimental group. Ultimately, it was agreed that all participants would accumulate steps through the health application "Light-hearted." The group's cumulative steps were reported every three days. At the end of intervention, if the goal of the activity was achieved, then the research project would make a donation to charity as promised."

5-ix) Describe use parameters

Describe use parameters (e.g., intended “doses” and optimal timing for use). Clarify what instructions or recommendations were given to the user, e.g., regarding timing, frequency, heaviness of use, if any, or was the intervention used ad libitum.

subitem not at all important

- 1 ○
- 2 ◉
- 3 ○
- 4 ○
- 5 ○

essential

ล้างสิ่งที่เลือก

Does your paper address subitem 5-ix?

Copy and paste relevant sections from the manuscript (include quotes in quotation marks "like this" to indicate direct quotes from your manuscript), or elaborate on this item by providing additional information not in the ms, or briefly explain why the item is not applicable/relevant for your study

"delivered daily through online platforms"

5-x) Clarify the level of human involvement

Clarify the level of human involvement (care providers or health professionals, also technical assistance) in the e-intervention or as co-intervention (detail number and expertise of professionals involved, if any, as well as “type of assistance offered, the timing and frequency of the support, how it is initiated, and the medium by which the assistance is delivered”. It may be necessary to distinguish between the level of human involvement required for the trial, and the level of human involvement required for a routine application outside of a RCT setting (discuss under item 21 – generalizability).

subitem not at all important

1 (selected)

2

3

4

5

essential

ล้างสิ่งที่เลือก

Does your paper address subitem 5-x?

Copy and paste relevant sections from the manuscript (include quotes in quotation marks "like this" to indicate direct quotes from your manuscript), or elaborate on this item by providing additional information not in the ms, or briefly explain why the item is not applicable/relevant for your study

คำตอบของคุณ

5-xi) Report any prompts/reminders used

Report any prompts/reminders used: Clarify if there were prompts (letters, emails, phone calls, SMS) to use the application, what triggered them, frequency etc. It may be necessary to distinguish between the level of prompts/reminders required for the trial, and the level of prompts/reminders for a routine application outside of a RCT setting (discuss under item 21 – generalizability).

subitem not at all important

1 ○

2 ○

3 ○

4 ○

5 ●

essential

ล้างสิ่งที่เลือก

Does your paper address subitem 5-xi? *

Copy and paste relevant sections from the manuscript (include quotes in quotation marks "like this" to indicate direct quotes from your manuscript), or elaborate on this item by providing additional information not in the ms, or briefly explain why the item is not applicable/relevant for your study

"While participants in the control group performed their regular routines, participants in the experimental group were exposed to 8-weeks intervention."
"Providing knowledge to increase awareness.
This part of the intervention lasted 2 weeks. This activity aimed to build knowledge and understanding of guidelines on PA for the intervention group. In the first week, participants were exposed to health education messages (infographic and video clips) sent through a health application "Light-hearted," and LINE. The messages covered PA self-assessment, the importance of PA, advantages, and disadvantages of the original behavior, as well as co-benefits of sufficient PA, and dramatic relief. In the second week, the health education message described the practice of various physical activities. This included information about alternatives to PA, and PA for the working-age population, both in the home/around the house, at work, and in public areas. The intention was to add a variety of options for PA. The video was produced by Social Marketing Team of XXXXXX, was in a public domain (XXXXXX YouTube channel) and free to be used. The videos contained messages on the causes of NCDs, shared experience and motivation for regular exercise."
"Health education messages were delivered through selected channels (i.e., Light-hearted and LINE application) to provide PA-related knowledge and create awareness of the importance of PA. In the first two weeks, various topics were delivered through LINE application and YouTube videos to improve participants' knowledge in PA, including the importance, co-benefit, choices of PA, and also guidelines practices for various PA."
"Creating Motivation.
A total of 6 weeks was spent to apply the Collective Action Theory, which was the main process hypothesized to drive behavior change in this study. Target groups set goals for PA together, with a time limit and group consensus as to when the goal was to be achieved. The Collective Action Theory prescribes both internal and external motivation. Both types of motivation were directly targeted by the intervention activity, and indirectly targeted through discussion and encouragement via group chat as an additional impetus to behavior change. The process started with setting group goals by using Zoom meetings to discuss PA goals in the entire experimental group. Ultimately, it was agreed that all participants would accumulate steps through the health application "Light-hearted." The group's cumulative steps were reported every three days. At the end of intervention, if the goal of the activity was achieved, then the research project would make a donation to charity as promised."
"In the 3rd-6th week, participants were invited to join ZOOM application to collectively setting up their goals. Motivational messages and reminders were sent throughout the 4 weeks through Light-hearted application to ensure participants' compliance."

5-xii) Describe any co-interventions (incl. training/support)

Describe any co-interventions (incl. training/support): Clearly state any interventions that are provided in addition to the targeted eHealth intervention, as ehealth intervention may not be designed as stand-alone intervention. This includes training sessions and support [1]. It may be necessary to distinguish between the level of training required for the trial, and the level of training for a routine application outside of a RCT setting (discuss under item 21 – generalizability.

subitem not at all important

1 (selected)

2

3

4

5

essential

ล้างสิ่งที่เลือก

Does your paper address subitem 5-xii? *

Copy and paste relevant sections from the manuscript (include quotes in quotation marks "like this" to indicate direct quotes from your manuscript), or elaborate on this item by providing additional information not in the ms, or briefly explain why the item is not applicable/relevant for your study

"subitem not at all important"

6a) Completely defined pre-specified primary and secondary outcome measures, including how and when they were assessed

Does your paper address CONSORT subitem 6a? *

Copy and paste relevant sections from the manuscript (include quotes in quotation marks "like this" to indicate direct quotes from your manuscript), or elaborate on this item by providing additional information not in the ms, or briefly explain why the item is not applicable/relevant for your study

"Eligible participants were screened according to the control variables, namely sex, age group, and primary occupation. The inclusion criteria were those who had insufficient physical activity as recommended by WHO and were in the second stage of behavior change (i.e., Contemplation)."

6a-i) Online questionnaires: describe if they were validated for online use and apply CHERRIES items to describe how the questionnaires were designed/deployed

If outcomes were obtained through online questionnaires, describe if they were validated for online use and apply CHERRIES items to describe how the questionnaires were designed/deployed [9].

subitem not at all important

1 (selected)

2

3

4

5

essential

ล้างสิ่งที่เลือก

Does your paper address subitem 6a-i?

Copy and paste relevant sections from manuscript text

"subitem not at all important"

6a-ii) Describe whether and how “use” (including intensity of use/dosage) was defined/measured/monitored

Describe whether and how “use” (including intensity of use/dosage) was defined/measured/monitored (logins, logfile analysis, etc.). Use/adoption metrics are important process outcomes that should be reported in any ehealth trial.

subitem not at all important

- 1 (selected)
- 2
- 3
- 4
- 5

essential

ล้างสิ่งที่เลือก

Does your paper address subitem 6a-ii?

Copy and paste relevant sections from manuscript text

"subitem not at all important"

6a-iii) Describe whether, how, and when qualitative feedback from participants was obtained

Describe whether, how, and when qualitative feedback from participants was obtained (e.g., through emails, feedback forms, interviews, focus groups).

subitem not at all important

1 ○

2 ◉

3 ○

4 ○

5 ○

essential

ล้างสิ่งที่เลือก

Does your paper address subitem 6a-iii?

Copy and paste relevant sections from manuscript text

"after the intervention finished by using Zoom meeting application"

6b) Any changes to trial outcomes after the trial commenced, with reasons

Does your paper address CONSORT subitem 6b? *

Copy and paste relevant sections from the manuscript (include quotes in quotation marks "like this" to indicate direct quotes from your manuscript), or elaborate on this item by providing additional information not in the ms, or briefly explain why the item is not applicable/relevant for your study

"Not changed trial outcomes after the trial commenced"

7a) How sample size was determined

NPT: When applicable, details of whether and how the clustering by care provides or centers was addressed

7a-i) Describe whether and how expected attrition was taken into account when calculating the sample size

Describe whether and how expected attrition was taken into account when calculating the sample size.

subitem not at all important

1 ○

2 ○

3 ○

4 ○

5 ◉

essential

ล้างสิ่งที่เลือก

Does your paper address subitem 7a-i?

Copy and paste relevant sections from manuscript title (include quotes in quotation marks "like this" to indicate direct quotes from your manuscript), or elaborate on this item by providing additional information not in the ms, or briefly explain why the item is not applicable/relevant for your study

"The population universe in this research is Thais adults who belong to the gen Y and who have access to the Internet, or a total of 17,644,290 people. The Gen Y population was defined as a group of the people those were born between 1981 and 2000 (22-26). This segment of the population is critical for the future development of the country, both in the labor market and in terms of the economy (insofar as the Gen Y group dominates the spending power of the country). This population was randomly sampled (with probability known) to provide information on Thailand Physical Activity Surveillance System. The prescribed sample size was calculated by considering the value alpha = 0.05, effect size = 0.5 (a medium effect size from the previous study), power = 0.95, and a 2-tail test. To reduce attrition due to withdrawal from the study or refusal to participate, the sample size was increased from the minimum prescribed number (54 persons), resulting in a total sample of 100 people, evenly divided into experimental and

7b) When applicable, explanation of any interim analyses and stopping guidelines

Does your paper address CONSORT subitem 7b? *

Copy and paste relevant sections from the manuscript (include quotes in quotation marks "like this" to indicate direct quotes from your manuscript), or elaborate on this item by providing additional information not in the ms, or briefly explain why the item is not applicable/relevant for your study

"No interim analyses and stopping guidelines"

8a) Method used to generate the random allocation sequence

NPT: When applicable, how care providers were allocated to each trial group

Does your paper address CONSORT subitem 8a? *

Copy and paste relevant sections from the manuscript (include quotes in quotation marks "like this" to indicate direct quotes from your manuscript), or elaborate on this item by providing additional information not in the ms, or briefly explain why the item is not applicable/relevant for your study

"Once the sample size was known, the researcher conducted random sampling to obtain the participants in the research by concealment of allocation sequences and participants do not know that who was assigned to experimental or control group as follows:
Step 1 Screening samples to participate in the experiment. The population universe was Thais of all genders and ages who had access to the Internet at the time of the survey. The sample was selected through multi-stage random sampling. For this study the population of interest was members of Gen Y. Eligible participants were screened according to the control variables, namely sex, age group, and primary occupation. The inclusion criteria were those who had insufficient physical activity as recommended by WHO and were in the second stage of behavior change (i.e., Contemplation), and who had access to the Internet at the time of the survey.
Step 2 Simple Random Sampling with replacement was used to randomly selected between experimental group and control group. At this stage, the prior random individual was assigned to experimental group, then another of its pair would be assigned to control group. This loop will be proceeded until all required sample were matches as planning.
Step 3 After the sampling process completed, the consent and voluntary participation were inquired. In case of sample rejection, the new random sample with the same

8b) Type of randomisation; details of any restriction (such as blocking and block size)

Does your paper address CONSORT subitem 8b? *

Copy and paste relevant sections from the manuscript (include quotes in quotation marks "like this" to indicate direct quotes from your manuscript), or elaborate on this item by providing additional information not in the ms, or briefly explain why the item is not applicable/relevant for your study

"In case of sample rejection, the new random sample with the same eligible and method was replaced."

9) Mechanism used to implement the random allocation sequence (such as sequentially numbered containers), describing any steps taken to conceal the sequence until interventions were assigned

Does your paper address CONSORT subitem 9? *

Copy and paste relevant sections from the manuscript (include quotes in quotation marks "like this" to indicate direct quotes from your manuscript), or elaborate on this item by providing additional information not in the ms, or briefly explain why the item is not applicable/relevant for your study

"Simple Random Sampling with replacement was used to randomly selected between experimental group and control group. At this stage, the prior random individual was assigned to experimental group, then another of its pair would be assigned to control group. This loop will be proceeded until all required sample were matches as planning."

10) Who generated the random allocation sequence, who enrolled participants, and who assigned participants to interventions

Does your paper address CONSORT subitem 10? *

Copy and paste relevant sections from the manuscript (include quotes in quotation marks "like this" to indicate direct quotes from your manuscript), or elaborate on this item by providing additional information not in the ms, or briefly explain why the item is not applicable/relevant for your study

"The researcher conducted random sampling to obtain the participants in the research by concealment of allocation sequences and participants do not know that who was assigned to experimental or control group."

11a) If done, who was blinded after assignment to interventions (for example, participants, care providers, those assessing outcomes) and how

NPT: Whether or not administering co-interventions were blinded to group assignment

11a-i) Specify who was blinded, and who wasn't

Specify who was blinded, and who wasn't. Usually, in web-based trials it is not possible to blind the participants [1, 3] (this should be clearly acknowledged), but it may be possible to blind outcome assessors, those doing data analysis or those administering co-interventions (if any).

subitem not at all important

1 ○

2 ○

3 ○

4 ○

5 ◉

essential

ล้างสิ่งที่เลือก

Does your paper address subitem 11a-i? *

Copy and paste relevant sections from the manuscript (include quotes in quotation marks "like this" to indicate direct quotes from your manuscript), or elaborate on this item by providing additional information not in the ms, or briefly explain why the item is not applicable/relevant for your study

"Single-blind assignment of subjects to experimental and control groups was employed."

11a-ii) Discuss e.g., whether participants knew which intervention was the “intervention of interest” and which one was the “comparator”

Informed consent procedures (4a-ii) can create biases and certain expectations - discuss e.g., whether participants knew which intervention was the “intervention of interest” and which one was the “comparator”.

subitem not at all important

1 (selected)

2

3

4

5

essential

ล้างสิ่งที่เลือก

Does your paper address subitem 11a-ii?

Copy and paste relevant sections from the manuscript (include quotes in quotation marks "like this" to indicate direct quotes from your manuscript), or elaborate on this item by providing additional information not in the ms, or briefly explain why the item is not applicable/relevant for your study

"If the intervention was successful in the experimental group, the intervention would also be offered to the control group participants"

11b) If relevant, description of the similarity of interventions

(this item is usually not relevant for ehealth trials as it refers to similarity of a placebo or sham intervention to a active medication/intervention)

Does your paper address CONSORT subitem 11b? *

Copy and paste relevant sections from the manuscript (include quotes in quotation marks "like this" to indicate direct quotes from your manuscript), or elaborate on this item by providing additional information not in the ms, or briefly explain why the item is not applicable/relevant for your study

"No relevant of similarity of interventions"

12a) Statistical methods used to compare groups for primary and secondary outcomes

NPT: When applicable, details of whether and how the clustering by care providers or centers was addressed

Does your paper address CONSORT subitem 12a? *

Copy and paste relevant sections from the manuscript (include quotes in quotation marks "like this" to indicate direct quotes from your manuscript), or elaborate on this item by providing additional information not in the ms, or briefly explain why the item is not applicable/relevant for your study

"To test the effectiveness of the model, the mean before-after values in the same group were compared by paired t-test, while the means between the two groups were compared by independent sample t-test."

12a-i) Imputation techniques to deal with attrition / missing values

Imputation techniques to deal with attrition / missing values: Not all participants will use the intervention/comparator as intended and attrition is typically high in ehealth trials. Specify how participants who did not use the application or dropped out from the trial were treated in the statistical analysis (a complete case analysis is strongly discouraged, and simple imputation techniques such as LOCF may also be problematic [4]).

subitem not at all important

1 (selected)

2

3

4

5

essential

ล้างสิ่งที่เลือก

Does your paper address subitem 12a-i? *

Copy and paste relevant sections from the manuscript (include quotes in quotation marks "like this" to indicate direct quotes from your manuscript), or elaborate on this item by providing additional information not in the ms, or briefly explain why the item is not applicable/relevant for your study

"No imputation techniques to deal with attrition"

12b) Methods for additional analyses, such as subgroup analyses and adjusted analyses

Does your paper address CONSORT subitem 12b? *

Copy and paste relevant sections from the manuscript (include quotes in quotation marks "like this" to indicate direct quotes from your manuscript), or elaborate on this item by providing additional information not in the ms, or briefly explain why the item is not applicable/relevant for your study

"Univariate analysis was used to describe the characteristics and distribution of the sample such as percentage, mean, median, and range. Bivariate analysis was used to test for significant association of variables and group-level differences."

X26) REB/IRB Approval and Ethical Considerations [recommended as subheading under "Methods"] (not a CONSORT item)

X26-i) Comment on ethics committee approval

subitem not at all important

1 ○

2 ○

3 ○

4 ○

5 ◉

essential

ล้างสิ่งที่เลือก

Does your paper address subitem X26-i?

Copy and paste relevant sections from the manuscript (include quotes in quotation marks "like this" to indicate direct quotes from your manuscript), or elaborate on this item by providing additional information not in the ms, or briefly explain why the item is not applicable/relevant for your study

"1. Conduct the research according to the approved protocol.
2. Conduct the informed consent process without coercion or undue influence, and provide the potential subjects sufficient time to consider whether or not to participate.
3. Use only the Consent Form bearing the IPSR-IRB Approval stamp.
4. Obtain approval of any changes in research activity before commencing and informed research participants about the changes for their consideration in pursuing the research.
5. Timely report to serious adverse events to IPSR-IRB and any new information that may adversely affect the safety of participants.
6. Provide IPSR-IRB the progress reports at least annually or as requested.

x26-ii) Outline informed consent procedures

Outline informed consent procedures e.g., if consent was obtained offline or online (how? Checkbox, etc.?), and what information was provided (see 4a-ii). See [6] for some items to be included in informed consent documents.

subitem not at all important

1 ○
2 ◉
3 ○
4 ○
5 ○

essential

ล้างสิ่งที่เลือก

Does your paper address subitem X26-ii?

Copy and paste relevant sections from the manuscript (include quotes in quotation marks "like this" to indicate direct quotes from your manuscript), or elaborate on this item by providing additional information not in the ms, or briefly explain why the item is not applicable/relevant for your study

"Participants provided their consent to be included in the study by click on the agreement box available in the Lime-survey web application. No personal information of the participant was collected or used in a way that would identify the data provider."

X26-iii) Safety and security procedures

Safety and security procedures, incl. privacy considerations, and any steps taken to reduce the likelihood or detection of harm (e.g., education and training, availability of a hotline)

subitem not at all important

- 1 (selected)
- 2
- 3
- 4
- 5

essential

ล้างสิ่งที่เลือก

Does your paper address subitem X26-iii?

Copy and paste relevant sections from the manuscript (include quotes in quotation marks "like this" to indicate direct quotes from your manuscript), or elaborate on this item by providing additional information not in the ms, or briefly explain why the item is not applicable/relevant for your study

"subitem not at all important"

RESULTS

13a) For each group, the numbers of participants who were randomly assigned, received intended treatment, and were analysed for the primary outcome

NPT: The number of care providers or centers performing the intervention in each group and the number of patients treated by each care provider in each center

Does your paper address CONSORT subitem 13a? *

Copy and paste relevant sections from the manuscript (include quotes in quotation marks "like this" to indicate direct quotes from your manuscript), or elaborate on this item by providing additional information not in the ms, or briefly explain why the item is not applicable/relevant for your study

"The remaining 80 subjects in the study comprised of 40 individuals each in the experimental and control groups."

13b) For each group, losses and exclusions after randomisation, together with reasons

Does your paper address CONSORT subitem 13b? (NOTE: Preferably, this is shown in a CONSORT flow diagram) *

Copy and paste relevant sections from the manuscript (include quotes in quotation marks "like this" to indicate direct quotes from your manuscript), or elaborate on this item by providing additional information not in the ms, or briefly explain why the item is not applicable/relevant for your study

"Of the 100 subjects, 8 left the study during the trial period and 12 were excluded because they declined to provide post-test data, yielding a 20% attrition rate." Because poor health condition acn infected COVID-19.

13b-i) Attrition diagram

Strongly recommended: An attrition diagram (e.g., proportion of participants still logging in or using the intervention/comparator in each group plotted over time, similar to a survival curve) or other figures or tables demonstrating usage/dose/engagement.

subitem not at all important

1 ○

2 ○

3 ○

4 ○

5 ◉

essential

ล้างสิ่งที่เลือก

Does your paper address subitem 13b-i?

Copy and paste relevant sections from the manuscript or cite the figure number if applicable (include quotes in quotation marks "like this" to indicate direct quotes from your manuscript), or elaborate on this item by providing additional information not in the ms, or briefly explain why the item is not applicable/relevant for your study

"Figure 2. Experimental and Control Groups in the Research."

14a) Dates defining the periods of recruitment and follow-up

Does your paper address CONSORT subitem 14a? *

Copy and paste relevant sections from the manuscript (include quotes in quotation marks "like this" to indicate direct quotes from your manuscript), or elaborate on this item by providing additional information not in the ms, or briefly explain why the item is not applicable/relevant for your study

"The intervention covered 8 weeks of activity (from August 22 to October 16, 2021)."

14a-i) Indicate if critical "secular events" fell into the study period

Indicate if critical "secular events" fell into the study period, e.g., significant changes in Internet resources available or "changes in computer hardware or Internet delivery resources"

subitem not at all important

- 1 (selected)
- 2
- 3
- 4
- 5

essential

ล้างสิ่งที่เลือก

Does your paper address subitem 14a-i?

Copy and paste relevant sections from the manuscript (include quotes in quotation marks "like this" to indicate direct quotes from your manuscript), or elaborate on this item by providing additional information not in the ms, or briefly explain why the item is not applicable/relevant for your study

"subitem not at all important"

14b) Why the trial ended or was stopped (early)

Does your paper address CONSORT subitem 14b? *

Copy and paste relevant sections from the manuscript (include quotes in quotation marks "like this" to indicate direct quotes from your manuscript), or elaborate on this item by providing additional information not in the ms, or briefly explain why the item is not applicable/relevant for your study

"The trial are not stopped early."

15) A table showing baseline demographic and clinical characteristics for each group

NPT: When applicable, a description of care providers (case volume, qualification, expertise, etc.) and centers (volume) in each group

Does your paper address CONSORT subitem 15? *

Copy and paste relevant sections from the manuscript (include quotes in quotation marks "like this" to indicate direct quotes from your manuscript), or elaborate on this item by providing additional information not in the ms, or briefly explain why the item is not applicable/relevant for your study

"The sample of the study comprised of Gen-Y population with similar age in both experimental (mean age 29.6, S.D. 5.52) and control (mean age 29.2, S.D. 6.48) group. In terms of sex, the proportion of females in both intervention and control group was slightly higher (60% and 55%, respectively) than males (40% and 45%, respectively). The proportion of sample in the experiment group who employed as office worker was slightly higher (45%) than their counterparts in the control group (35%). There was no statistically significant difference for general characteristics, health factors, perceptions about PA, and level of moderate-to-vigorous PA (MVPA) at baseline. Prior to the intervention, individuals in the experimental group collected a slightly higher average cumulative MVPA (62.15, S.D.±89.38 mins per week) than those in the control group (43.55, S.D.± 62.15 mins per week). Nevertheless, there was no significant difference in the PA level between the two groups (P =.29)."

15-i) Report demographics associated with digital divide issues

In ehealth trials it is particularly important to report demographics associated with digital divide issues, such as age, education, gender, social-economic status, computer/Internet/ehealth literacy of the participants, if known.

subitem not at all important

1 ◉

2 ○

3 ○

4 ○

5 ○

essential

ล้างสิ่งที่เลือก

Does your paper address subitem 15-i? *

Copy and paste relevant sections from the manuscript (include quotes in quotation marks "like this" to indicate direct quotes from your manuscript), or elaborate on this item by providing additional information not in the ms, or briefly explain why the item is not applicable/relevant for your study

"In the study comprised of 80 Gen Y population only"

16) For each group, number of participants (denominator) included in each analysis and whether the analysis was by original assigned groups

16-i) Report multiple “denominators” and provide definitions

Report multiple “denominators” and provide definitions: Report N’s (and effect sizes) “across a range of study participation [and use] thresholds” [1], e.g., N exposed, N consented, N used more than x times, N used more than y weeks, N participants “used” the intervention/comparator at specific pre-defined time points of interest (in absolute and relative numbers per group). Always clearly define “use” of the intervention.

subitem not at all important

1 ○

2 ○

3 ○

4 ○

5 ◉

essential

ล้างสิ่งที่เลือก

Does your paper address subitem 16-i? *

Copy and paste relevant sections from the manuscript (include quotes in quotation marks "like this" to indicate direct quotes from your manuscript), or elaborate on this item by providing additional information not in the ms, or briefly explain why the item is not applicable/relevant for your study

"With 80% of general compliance rate and 63% success rate, yielding the effect size of the intervention at 28% [effect size = 27.5, number needed to treat (NNT) = 3.64]."

16-ii) Primary analysis should be intent-to-treat

Primary analysis should be intent-to-treat, secondary analyses could include comparing only “users”, with the appropriate caveats that this is no longer a randomized sample (see 18-i).

subitem not at all important

1 (selected)

2

3

4

5

essential

ล้างสิ่งที่เลือก

Does your paper address subitem 16-ii?

Copy and paste relevant sections from the manuscript (include quotes in quotation marks "like this" to indicate direct quotes from your manuscript), or elaborate on this item by providing additional information not in the ms, or briefly explain why the item is not applicable/relevant for your study

"subitem not at all important"

17a) For each primary and secondary outcome, results for each group, and the estimated effect size and its precision (such as 95% confidence interval)

Does your paper address CONSORT subitem 17a? *

Copy and paste relevant sections from the manuscript (include quotes in quotation marks "like this" to indicate direct quotes from your manuscript), or elaborate on this item by providing additional information not in the ms, or briefly explain why the item is not applicable/relevant for your study

"For bivariate analysis was used to test for significant association of variables and group-level differences. Before participating in the trial, both sample groups were at the second stage of behavior change (i.e., contemplation). According to Transtheoretical Model of Behavioral Change (TTM), contemplation means the individuals intend to change their behavior (into a positive one) within the next 6 months. Upon completing an 8-weeks intervention, 63% of participants in the experimental group and 35% participants in the control group improved their level of behavior to the 4th stage (i.e., action) by reporting an improvement in their PA for less than 6 month. While most of participants in the experimental group has progressed to action, 50% of participants in the control group remained in the contemplation stage. The findings suggest that the intervention helped those in the experimental group to advance in their stage of behavioral readiness, i.e., to take action (P =.012) ($\chi 2$ = 6.05)."
"To test the effectiveness of the intervention, independent sample t-test was performed to compare the duration of MVPA (minutes) between participants in the experimental and control group. After being exposed to the intervention, participants in the intervention group collected a higher cumulative minute of MVPA weekly (283 mins) than those in the control group (164 mins), and this was statistically significant at the .05 level (t = 2.19)."
"The effectiveness of the intervention also could be observed from the increase in the cumulative minutes of MVPA collected by participants in the experimental group. The analysis considered the difference in duration of PA before and after participating in the study intervention among members of the experimental group using the paired samples t-test statistic. The analysis found that the mean duration of PA before and after participating in the intervention among the experimental group was significantly different at the P < .001 level (t = 4.73). PA increased by 221.38 minutes, suggesting that the model can significantly increase the PA of Gen Y persons exposed to the intervention."
"In the baseline, 10% (4/40) of participants in the experimental group and 8% in the control group met the recommended level of MVPA for adults. After being exposed to the study intervention, the experimental group had more sufficient PA than their counterparts in the control group, and that difference was statistically significant (P =.032). Over two-fifths, (63%) of members in the experimental group had adequate PA compared to only slightly over one-third of the control group (35%). With 80% of general compliance rate and 63% success rate, yielding the effect size of the intervention at 28% [effect size = 27.5, number needed to treat (NNT) = 3.64]. Those who were exposed to the full package of interventions and comply (96%) recorded positive change, having sufficient PA, and that improvement was statistically significant (P <.001) ($\chi 2$ = 31.922). Males, adults aged 30-39 years and employed in non-formal sector are more likely to

17a-i) Presentation of process outcomes such as metrics of use and intensity of use

In addition to primary/secondary (clinical) outcomes, the presentation of process outcomes such as metrics of use and intensity of use (dose, exposure) and their operational definitions is critical. This does not only refer to metrics of attrition (13-b) (often a binary variable), but also to more continuous exposure metrics such as "average session length". These must be accompanied by a technical description how a metric like a "session" is defined (e.g., timeout after idle time) [1] (report under item 6a).

subitem not at all important

1 (selected)

2

3

4

5

essential

ล้างสิ่งที่เลือก

Does your paper address subitem 17a-i?

Copy and paste relevant sections from the manuscript (include quotes in quotation marks "like this" to indicate direct quotes from your manuscript), or elaborate on this item by providing additional information not in the ms, or briefly explain why the item is not applicable/relevant for your study

"subitem not at all important"

17b) For binary outcomes, presentation of both absolute and relative effect sizes is recommended

Does your paper address CONSORT subitem 17b? *

Copy and paste relevant sections from the manuscript (include quotes in quotation marks "like this" to indicate direct quotes from your manuscript), or elaborate on this item by providing additional information not in the ms, or briefly explain why the item is not applicable/relevant for your study

"In the baseline, 10% (4/40) of participants in the experimental group and 8% in the control group met the recommended level of MVPA for adults. After being exposed to the study intervention, the experimental group had more sufficient PA than their counterparts in the control group, and that difference was statistically significant (P =.032). Over two-fifths, (63%) of members in the experimental group had adequate PA compared to only slightly over one-third of the control group (35%). With 80% of general compliance rate and 63% success rate, yielding the effect size of the intervention at 28% [effect size = 27.5, number needed to treat (NNT) = 3.64]. Those who were exposed to the full package of interventions and comply (96%) recorded positive change, having sufficient PA, and that improvement was statistically significant (P <.001) ($\chi 2$ = 31.922). Males, adults aged 30-39 years and employed in non-formal sector are more likely to comply to the intervention compared to females, younger adults (20-29 years) and unemployed or office workers."

18) Results of any other analyses performed, including subgroup analyses and adjusted analyses, distinguishing pre-specified from exploratory

Does your paper address CONSORT subitem 18? *

Copy and paste relevant sections from the manuscript (include quotes in quotation marks "like this" to indicate direct quotes from your manuscript), or elaborate on this item by providing additional information not in the ms, or briefly explain why the item is not applicable/relevant for your study

"No other analyses performed, including subgroup analyses"

18-i) Subgroup analysis of comparing only users

A subgroup analysis of comparing only users is not uncommon in ehealth trials, but if done, it must be stressed that this is a self-selected sample and no longer an unbiased sample from a randomized trial (see 16-iii).

subitem not at all important

1 ○

2 ○

3 ○

4 ○

5 ◉

essential

ล้างสิ่งที่เลือก

Does your paper address subitem 18-i?

Copy and paste relevant sections from the manuscript (include quotes in quotation marks "like this" to indicate direct quotes from your manuscript), or elaborate on this item by providing additional information not in the ms, or briefly explain why the item is not applicable/relevant for your study

"The analysis considered the difference in duration of PA before and after participating in the study intervention among members of the experimental group using the paired samples t-test statistic. The analysis found that the mean duration of PA before and after participating in the intervention among the experimental group was significantly different at the $P < .001$ level ($t = 4.73$). PA increased by 221.38 minutes, suggesting that the model can significantly increase the PA of Gen Y persons exposed to the

19) All important harms or unintended effects in each group

(for specific guidance see CONSORT for harms)

Does your paper address CONSORT subitem 19? *

Copy and paste relevant sections from the manuscript (include quotes in quotation marks "like this" to indicate direct quotes from your manuscript), or elaborate on this item by providing additional information not in the ms, or briefly explain why the item is not applicable/relevant for your study

"It is noteworthy that members in the control group also reported an increase in PA during the trial period, but not to the same level as those in the experimental group. This could be due to external factors which were affecting Gen Y persons generally in society, separate from any explicit intervention. In addition, perhaps the mere act of inviting those members of the control group to participate in the study may have been motivational in and of itself (39). The members of the control group may have become more self-aware of their PA level during the pre-test phase, and took steps to improve their PA on their own initiative. Or perhaps, the changes in the post-test PA of both groups were due to less restriction measures with limited facility opening."

19-i) Include privacy breaches, technical problems

Include privacy breaches, technical problems. This does not only include physical “harm” to participants, but also incidents such as perceived or real privacy breaches [1], technical problems, and other unexpected/unintended incidents. “Unintended effects” also includes unintended positive effects [2].

subitem not at all important

1 (selected)

2

3

4

5

essential

ล้างสิ่งที่เลือก

Does your paper address subitem 19-i?

Copy and paste relevant sections from the manuscript (include quotes in quotation marks "like this" to indicate direct quotes from your manuscript), or elaborate on this item by providing additional information not in the ms, or briefly explain why the item is not applicable/relevant for your study

"subitem not at all important"

19-ii) Include qualitative feedback from participants or observations from staff/researchers

Include qualitative feedback from participants or observations from staff/researchers, if available, on strengths and shortcomings of the application, especially if they point to unintended/unexpected effects or uses. This includes (if available) reasons for why people did or did not use the application as intended by the developers.

subitem not at all important

1 ○

2 ◉

3 ○

4 ○

5 ○

essential

ล้างสิ่งที่เลือก

Does your paper address subitem 19-ii?

Copy and paste relevant sections from the manuscript (include quotes in quotation marks "like this" to indicate direct quotes from your manuscript), or elaborate on this item by providing additional information not in the ms, or briefly explain why the item is not applicable/relevant for your study

"Feedback from participants, researchers received suggestions from the experimental group for application development after the intervention finished by using Zoom meeting application, such as the frequency of transmission notifications, group chat not notified, and should add varieties of physical activity selection functions, etc., because it will be more interesting to the application."

DISCUSSION

22) Interpretation consistent with results, balancing benefits and harms, and considering other relevant evidence

NPT: In addition, take into account the choice of the comparator, lack of or partial blinding, and unequal expertise of care providers or centers in each group

22-i) Restate study questions and summarize the answers suggested by the data, starting with primary outcomes and process outcomes (use)

Restate study questions and summarize the answers suggested by the data, starting with primary outcomes and process outcomes (use).

subitem not at all important

1 ○

2 ○

3 ○

4 ○

5 ◉

essential

ล้างสิ่งที่เลือก

Does your paper address subitem 22-i? *

Copy and paste relevant sections from the manuscript (include quotes in quotation marks "like this" to indicate direct quotes from your manuscript), or elaborate on this item by providing additional information not in the ms, or briefly explain why the item is not applicable/relevant for your study

"Online group-based activity shown its effectiveness in improving PA of Gen Y in the intervention group. The COVID-19 pandemic has affected the PA of the population globally. This study found that applying online group-based activity intervention can help in regenerating PA of Gen Y by considering the context of their lifestyle and habits. The online group-based activity was able to significantly boost the PA level of behavior of members of the experimental group and shift from contemplation stage to action. Over three in five members of the experimental group (63%) have successfully changed their behavior by taking an action and improve their PA. The findings of this study indicated that the prototype model of online group-based activity was appropriate for GenY as it fits their characteristics and lifestyle. The health information packages in the intervention were beneficial for GenY in accelerating their behavior changes, as it contained a series of practical knowledge and examples on PA during COVID-19 pandemic containment periods. The application of Trans-Theoretical Model of Changes also helped in ensuring the intervention match with the stage of behavior that an individual member may have differences."

22-ii) Highlight unanswered new questions, suggest future research

Highlight unanswered new questions, suggest future research.

subitem not at all important

- 1 ○
- 2 ○
- 3 ○
- 4 ◉
- 5 ○

essential

ล้างสิ่งที่เลือก

Does your paper address subitem 22-ii?

Copy and paste relevant sections from the manuscript (include quotes in quotation marks "like this" to indicate direct quotes from your manuscript), or elaborate on this item by providing additional information not in the ms, or briefly explain why the item is not applicable/relevant for your study

คำตอบของคุณ

20) Trial limitations, addressing sources of potential bias, imprecision, and, if relevant, multiplicity of analyses

20-i) Typical limitations in ehealth trials

Typical limitations in ehealth trials: Participants in ehealth trials are rarely blinded. Ehealth trials often look at a multiplicity of outcomes, increasing risk for a Type I error. Discuss biases due to non-use of the intervention/usability issues, biases through informed consent procedures, unexpected events.

subitem not at all important

1 ○

2 ○

3 ○

4 ○

5 ◉

essential

ล้างสิ่งที่เลือก

Does your paper address subitem 20-i? *

Copy and paste relevant sections from the manuscript (include quotes in quotation marks "like this" to indicate direct quotes from your manuscript), or elaborate on this item by providing additional information not in the ms, or briefly explain why the item is not applicable/relevant for your study

"the package of interventions was not assessed for efficacy over time after the trial period was complete. This makes it impossible to track the persistence of the recorded PA gains in the intervention group. In addition, this model was designed to be consistent with the Gen Y lifestyle. To expand the intervention in other population age groups, it would be necessary to take into account the prevailing stage of behavior and way of life of that population segment. In addition, the findings from this study are short-term. Creating profound behavior change to the 4th Stage (action), then on to the 5th Stage (maintenance) takes extensive time and a greater range and intensity of interventions than were possible in this study. As a result, there is no guarantee that the observed improvements in behavior of the experimental group were sustained for any length of time after the end of the trial. One more limitation is the measurement of physical activity is objective measurement not subjective measurement."

21) Generalisability (external validity, applicability) of the trial findings

NPT: External validity of the trial findings according to the intervention, comparators, patients, and care providers or centers involved in the trial

21-i) Generalizability to other populations

Generalizability to other populations: In particular, discuss generalizability to a general Internet population, outside of a RCT setting, and general patient population, including applicability of the study results for other organizations

subitem not at all important

1 (selected)

2

3

4

5

essential

ล้างสิ่งที่เลือก

Does your paper address subitem 21-i?

Copy and paste relevant sections from the manuscript (include quotes in quotation marks "like this" to indicate direct quotes from your manuscript), or elaborate on this item by providing additional information not in the ms, or briefly explain why the item is not applicable/relevant for your study

"subitem not at all important"

21-ii) Discuss if there were elements in the RCT that would be different in a routine application setting

Discuss if there were elements in the RCT that would be different in a routine application setting (e.g., prompts/reminders, more human involvement, training sessions or other co-interventions) and what impact the omission of these elements could have on use, adoption, or outcomes if the intervention is applied outside of a RCT setting.

subitem not at all important

1 ○

2 ○

3 ◉

4 ○

5 ○

essential

ล้างสิ่งที่เลือก

Does your paper address subitem 21-ii?

Copy and paste relevant sections from the manuscript (include quotes in quotation marks "like this" to indicate direct quotes from your manuscript), or elaborate on this item by providing additional information not in the ms, or briefly explain why the item is not applicable/relevant for your study

"The elements in the RCT that would be different is Group chat function."

OTHER INFORMATION

23) Registration number and name of trial registry

Does your paper address CONSORT subitem 23? *

Copy and paste relevant sections from the manuscript (include quotes in quotation marks "like this" to indicate direct quotes from your manuscript), or elaborate on this item by providing additional information not in the ms, or briefly explain why the item is not applicable/relevant for your study

"The study protocol was reviewed and approved by the Research Ethics Committee of the Institute for Population and Social Research of Mahidol University on April 28, 2021 (project code COA No. 2021/03-048) and registered on the Thai Clinical Trials Registry (TCTR) number TCTR20211101005."

24) Where the full trial protocol can be accessed, if available

Does your paper address CONSORT subitem 24? *

Cite a Multimedia Appendix, other reference, or copy and paste relevant sections from the manuscript (include quotes in quotation marks "like this" to indicate direct quotes from your manuscript), or elaborate on this item by providing additional information not in the ms, or briefly explain why the item is not applicable/relevant for your study

"Full trial protocol is not online available untill paper will be publish"

25) Sources of funding and other support (such as supply of drugs), role of funders

Does your paper address CONSORT subitem 25? *

Copy and paste relevant sections from the manuscript (include quotes in quotation marks "like this" to indicate direct quotes from your manuscript), or elaborate on this item by providing additional information not in the ms, or briefly explain why the item is not applicable/relevant for your study

"the support from the Biomed lab, the health application “Light-hearted” from Popmed Co. Ltd., and the Department of Biomedical Engineering, Faculty of Engineering, Mahidol University, Thailand"

X27) Conflicts of Interest (not a CONSORT item)

X27-i) State the relation of the study team towards the system being evaluated

In addition to the usual declaration of interests (financial or otherwise), also state the relation of the study team towards the system being evaluated, i.e., state if the authors/evaluators are distinct from or identical with the developers/sponsors of the intervention.

subitem not at all important

1 ○

2 ○

3 ○

4 ○

5 ◉

essential

ล้างสิ่งที่เลือก

Does your paper address subitem X27-i?

Copy and paste relevant sections from the manuscript (include quotes in quotation marks "like this" to indicate direct quotes from your manuscript), or elaborate on this item by providing additional information not in the ms, or briefly explain why the item is not applicable/relevant for your study

"The authors declare that they have no competing interests."

About the CONSORT EHEALTH checklist

As a result of using this checklist, did you make changes in your manuscript? *

- ○ yes, major changes
- ◉ yes, minor changes
- ○ no

What were the most important changes you made as a result of using this checklist?

"Future studies in the Discussion part, and feedback from participants in the Discussion part"

How much time did you spend on going through the checklist INCLUDING making changes in your manuscript *

"We spend on going through the checklist including making changes in the manuscript about 1 week."

As a result of using this checklist, do you think your manuscript has improved? *

- ◉ yes
- ○ no
- ○ อื่นๆ: ________

Would you like to become involved in the CONSORT EHEALTH group?

This would involve for example becoming involved in participating in a workshop and writing an "Explanation and Elaboration" document

- ○ yes
- ◉ no
- ○ อื่นๆ:

ล้างสิ่งที่เลือก

Any other comments or questions on CONSORT EHEALTH

คำตอบของคุณ

STOP - Save this form as PDF before you click submit

To generate a record that you filled in this form, we recommend to generate a PDF of this page (on a Mac, simply select "print" and then select "print as PDF") before you submit it.

When you submit your (revised) paper to JMIR, please upload the PDF as supplementary file.

Don't worry if some text in the textboxes is cut off, as we still have the complete information in our database. Thank you!

Final step: Click submit !

Click submit so we have your answers in our database!

ส่ง

ล้างแบบฟอร์ม

ห้ามส่งรหัสผ่านใน Google ฟอร์ม

เนื้อหานี้มิได้ถูกสร้างขึ้นหรือรับรองโดย Google รายงานการละเมิด - ข้อกำหนดในการให้บริการ - นโยบายความเป็นส่วนตัว

Google ฟอร์ม
